# Gaussian Multiuser Wiretap Channels in the Presence of a Jammer-Aided Eavesdropper †

**DOI:** 10.3390/e24111595

**Published:** 2022-11-02

**Authors:** Rémi A. Chou, Aylin Yener

**Affiliations:** 1 Department of Electrical Engineering and Computer Science, Wichita State University, Wichita, KS 67260, USA; 2 Department of Electrical and Computer Engineering, The Ohio State University, Columbus, OH 43210, USA

**Keywords:** Gaussian wiretap channel, Gaussian multiple-access wiretap channel, Gaussian broadcast wiretap channel, jamming, secure communication

## Abstract

This paper considers secure communication in the presence of an eavesdropper and a malicious jammer. The jammer is assumed to be oblivious of the communication signals emitted by the legitimate transmitter(s) but can employ any jamming strategy subject to a given power constraint and shares her jamming signal with the eavesdropper. Four such models are considered: (i) the Gaussian point-to-point wiretap channel; (ii) the Gaussian multiple-access wiretap channel; (iii) the Gaussian broadcast wiretap channel; and (iv) the Gaussian symmetric interference wiretap channel. The use of pre-shared randomness between the legitimate users is not allowed in our models. Inner and outer bounds are derived for these four models. For (i), the secrecy capacity is obtained. For (ii) and (iv) under a degraded setup, the optimal secrecy sum-rate is characterized. Finally, for (iii), ranges of model parameter values for which the inner and outer bounds coincide are identified.

## 1. Introduction

Consider secure communication over wireless channels between legitimate parties in the presence of an eavesdropper and a malicious jammer. The jammer is assumed to be oblivious of the legitimate users’ communication but can employ any jamming strategy subject to a given power constraint. Consequently, the main channel between the legitimate users is arbitrarily varying [1]. Unlike most works that consider arbitrarily varying channels, however, pre-shared randomness is not available to the legitimate users in our scenario. Additionally, the jammer shares her jamming signal with the eavesdropper who can thus perfectly cancel the effect of the jamming signal on her channel. In this paper, we study the fundamental limits of secure communication rates in the presence of such a jammer-aided eavesdropper over four Gaussian wiretap channel models: the Gaussian wiretap channel [2], the Gaussian multiple-access wiretap channel [3], the Gaussian broadcast wiretap channel [4], and the Gaussian symmetric interference wiretap channel.

### 1.1. Contributions

Our contributions are summarized as follows.

- For secure communication over Gaussian point-to-point, multiple-access, broadcast, and symmetric interference wiretap channels in the presence of a jammer-aided eavesdropper as described above, we determine inner and outer bounds on the secrecy capacity region.
- We show that our bounds are tight for the point-to-point setting, tight for sum-rates for the multiple-access and interference settings under degraded setups, and tight for some ranges of model parameter values for the broadcast setting.

Our main strategy to handle our multiuser settings is to reduce the problem to single-user coding. Previous known techniques for such a reduction, such as rate-splitting [5] and successive cancellation decoding [5] [Appendix C], that have been developed for multiple-access settings without security constraints, do not easily apply to wiretap channel models. These techniques consist in achieving the corner points of achievability regions that can be described by polymatroids whose corner points have *positive components*. However, regions described by polymatroids whose corner points have *negative components*, as in our wiretap channel models, prevent the applications of these techniques. We overcome this roadblock by proposing novel time-sharing strategies coupled with appropriate secret-key exchanges between the legitimate users. As seen in the proofs of our results, eavesdropping and arbitrary jamming are not easy to decouple in the secrecy analysis. In particular, the analysis of the secrecy in our proposed model does not follow from a standard secrecy analysis in the absence of jamming, as we need to consider (i) codewords uniformly distributed over spheres, which we use to handle an arbitrarily varying main channel; and (ii) block-Markov coding and specific time-sharing strategies (to allow the reduction of multiuser coding to single-user coding) which create inter-dependencies between coding blocks. Note that our achievability schemes also rely on point-to-point codes developed in [1]. One of the benefits of reducing multiuser coding to point-to-point coding techniques is that despite the fact that our setting involves multiple transmitters and an arbitrarily varying channel between the legitimate users, *pre-shared randomness among the legitimate users will not be needed in our achievability schemes*. Our strategy for the converse consists of reducing the problem of determining a converse for our model to the problem of determining a converse for a related model in the absence of a jammer.

### 1.2. Related Works

Related works that consider simultaneous eavesdropping and oblivious jamming threats for the point-to-point discrete memoryless wiretap channel include [6,7,8,9,10,11]. The proof techniques used in these references to obtain security, such as random binning [12,13], resolvability/soft covering [10,14,15], or typicality arguments, are challenging to apply to a Gaussian setting in the absence of shared randomness at the legitimate user. Specifically, for the Gaussian point-to-point channel in the presence of an adversary that arbitrarily jams [1], the only known coding mechanism to obtain reliability in the absence of pre-shared randomness relies on codewords uniformly drawn on a unit sphere [1], which are challenging to integrate with the above techniques to obtain security because their components are not independent and identically distributed.

Another line of work [16] considers Gaussian channel models where the eavesdropper channel can vary arbitrarily, but the main channel is not. The setting considered in the present paper, where the main channel between the legitimate users is arbitrarily varying, prevents the use of analyses similar to those in [16] for the same reasons described above.

Several other works have considered continuous channel models, including the Gaussian MIMO wiretap channel [17], the Gaussian multiple-access wiretap channel [18], where deviating users can be viewed as active adversary, and continuous point-to-point wiretap channels [19,20], where the adversary can choose between eavesdropping or jamming. These references differ from the above-mentioned references on arbitrarily varying channels as they assume a specific signaling strategy for the jammer.

Finally, note that for point-to-point channels, stronger jamming strategies that depend on the signals of the legitimate transmitters have been studied in [21,22,23].

### 1.3. Organization of the Paper

The remainder of the paper is organized as follows. We describe the models in Section 2. We present our results for the Gaussian point-to-point wiretap channel, the Gaussian multiple-access wiretap channel, the Gaussian broadcast wiretap channel, and the Gaussian symmetric interference wiretap channel in Section 3, Section 4, Section 5 and Section 6, respectively. We discuss in Section 4.2 a way to avoid, at least for some channel parameters, time-sharing for the multiple-access setting. We also discuss in Section 4.3 an extension of the multiple-access setting to more than two transmitters. We detail the proofs for the multiple-access setting in Section 7 and Section 8. We end the paper with concluding remarks in Section 9.

## 2. Problem Statement

### 2.1. Notation

For a,b∈R, define ⟦a,b⟧≜[⌊a⌋,⌈b⌉]∩N, ]a,b[≜[a,b]∖{a,b}, ]a,b]≜[a,b]∖{a}, and [a,b[≜[a,b]∖{b}. The components of a vector, $X^{n}$, of size n∈N, are denoted by subscripts, i.e., $X^{n}≜\left(X_{1},X_{2},\ldots ,X_{n}\right)$. For x∈R, define ${\left[x\right]}^{+}≜\max\left(0,x\right)$. The notation x↦y describes a function that associates *y* to *x* when the domain and the image of the function are clear from the context. The power set of a finite set S is denoted by $2^{S}$. The convex hull of a set S is denoted by Conv(S). Unless specified otherwise, capital letters designate random variables, whereas lowercase letters designate realizations of associated random variables, e.g., *x* is a realization of the random variable *X*. For $R\in R_{+}$, $B_{0}^{n}\left(R\right)$ denotes the ball of radius *R* centered in 0 in $R^{n}$ under the Euclidian norm.

### 2.2. Gaussian Multiuser Wiretap Channel in the Presence of a Jammer-Aided Eavesdropper

Consider the Gaussian memoryless wiretap channel model with two transmitters and two legitimate receivers

(1a) $$\begin{array}{cc} Y_{1}^{n} & ≜\sqrt{g_{11}}X_{1}^{n}+\sqrt{g_{12}}X_{2}^{n}+\sqrt{g_{13}}S^{n}+N_{1}^{n}, \end{array}$$

(1b) $$\begin{array}{cc} Y_{2}^{n} & ≜\sqrt{g_{21}}X_{1}^{n}+\sqrt{g_{22}}X_{2}^{n}+\sqrt{g_{23}}S^{n}+N_{2}^{n}, \end{array}$$

(1c) $$\begin{array}{cc} Z^{n} & ≜\sqrt{h_{1}}X_{1}^{n}+\sqrt{h_{2}}X_{2}^{n}+N_{Z}^{n}, \end{array}$$

where $Y_{1}^{n}$, $Y_{2}^{n}$ are the channel outputs observed by the legitimate receivers, and $Z^{n}$ is the channel output observed by the eavesdropper. For l∈{1,2}, $X_{l}^{n}$ is the signal emitted by Transmitter *l* satisfying the power constraint $∥X_{l}^{n}{∥}^{2}≜\sum_{i=1}^{n}{\left(X_{l}\right)}_{i}^{2}\leq n\Gamma_{l}$, $S^{n}$ is an arbitrary jamming sequence transmitted by the jammer that is oblivious of the communication of the legitimate users and satisfies the power constraint $∥S^{n}{∥}^{2}≜\sum_{i=1}^{n}S_{i}^{2}\leq n\Lambda$, and $N_{Y_{1}}^{n}$, $N_{Y_{2}}^{n}$, $N_{Z}^{n}$ are sequences of independent and identically distributed Gaussian noise with variances $\sigma_{1}^{2}$, $\sigma_{2}^{2}$, $\sigma_{Z}^{2}$, respectively. The channel coefficients $g_{11}$, $g_{12}$, $g_{13}$, $g_{21}$, $g_{22}$, $g_{23}$, $h_{1}$, $h_{2}$ are fixed and known to all parties. Note that we assume that the jammer helps the eavesdropper by sharing her jamming sequence, which allows the eavesdropper to perfectly cancel $S^{n}$ from $Z^{n}$. Coding schemes and achievable rates are defined as follows.

**Definition** **1.** *Let n,k∈N. A $\left(2^{nR_{1}},2^{nR_{2}},n,k\right)$ code $C_{n}$ consists, for each block j∈⟦1,k⟧, of*

- *Two message sets $M_{l}^{\left(j\right)}≜⟦1,2^{nR_{l}^{\left(j\right)}}⟧$, l∈{1,2};*
- *Two stochastic encoders, $e_{l}^{\left(j\right)}:M_{l}^{\left(j\right)}\to B_{0}^{n}\left(\sqrt{n\Gamma_{l}}\right)$, l∈{1,2};*
- *Two decoders, $g_{l}^{\left(j\right)}:R^{n}\to M_{l}^{\left(j\right)}$, l∈{1,2};*

*where for any l∈{1,2}, $R_{l}=\frac{1}{k}\sum_{j=1}^{k}R_{l}^{\left(j\right)}$, and operates as follows. For each block j∈⟦1,k⟧, transmitter l∈{1,2} encodes with $e_{l}^{\left(j\right)}$ a uniformly distributed message $M_{l}^{\left(j\right)}\in M_{l}^{\left(j\right)}$ to a codeword of length n, which is sent to the legitimate receiver over the channel described by Equation (1a), Equation (1b), Equation (1c) with the power constraint nΛ for the jamming signal $S_{i}^{n}$. Note that all the power constraints at the transmitters and the jammer hold for a given transmission block of length n, which is relevant when the power constraints hold within any time window corresponding to n channel uses. Then, the legitimate receiver l∈{1,2} forms an estimate ${\hat{M}}_{l}^{\left(j\right)}≜g_{l}^{\left(j\right)}\left(Y_{l}^{n}\right)$ of the message $M_{l}^{\left(j\right)}$. We define $\hat{M}≜{\left({\hat{M}}_{1}^{\left(j\right)},{\hat{M}}_{2}^{\left(j\right)}\right)}_{j\in ⟦1,k⟧}$, $M≜{\left(M_{1}^{\left(j\right)},M_{2}^{\left(j\right)}\right)}_{j\in ⟦1,k⟧}$, $S≜{\left(S_{i}^{n}\right)}_{i\in ⟦1,k⟧}$, and $S≜\{{\left(S_{i}^{n}\right)}_{i\in ⟦1,k⟧}:∥S_{i}^{n}{∥}^{2}\leq n\Lambda ,\forall i\in ⟦1,k⟧\}$.*

**Definition** **2.** *A rate pair $\left(R_{1},R_{2}\right)$ is achievable, if there exists a sequence of $\left(2^{nR_{1}},2^{nR_{2}},n,k\right)$ codes such that*

(2a) $$\begin{array}{cc} \lim_{n\to \infty }\underset{S\in S}{\sup}P\left[\hat{M}\neq M\right]=0 & \;\left(\mathrm{reliability}\right), \end{array}$$

(2b) $$\begin{array}{cc} \lim_{n\to \infty }\frac{1}{nk}H\left(M|Z^{kn}\right)\geq R_{1}+R_{2} & \;\left(\mathrm{equivocation}\right). \end{array}$$

### 2.3. Special Case 1: The Gaussian Wiretap Channel in the Presence of a Jammer-Aided Eavesdropper

**Figure 1:**
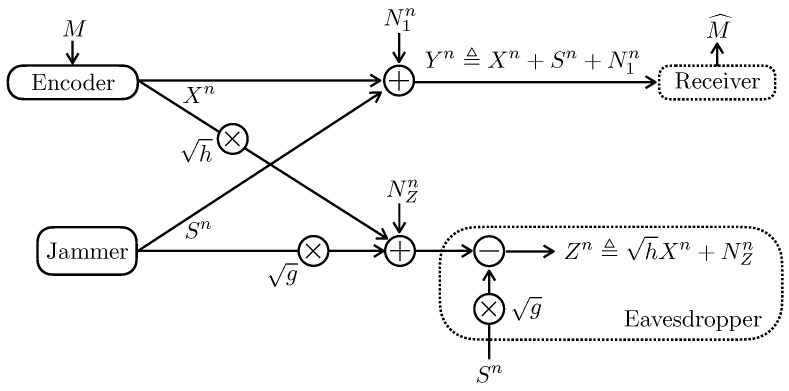
The Gaussian wiretap channel in the presence of a jammer-aided eavesdropper.

Assume that the two transmitters are colocated and the two receivers are colocated in Section 2.2. More specifically, as depicted in Figure 1, the channel model of Section 2.2 becomes

(3a) $$\begin{array}{cc} Y^{n} & ≜X^{n}+S^{n}+N_{1}^{n}, \end{array}$$

(3b) $$\begin{array}{cc} Z^{n} & ≜\sqrt{h}X^{n}+N_{Z}^{n}, \end{array}$$

where $\sigma_{1}^{2}=\sigma_{Z}^{2}=1$. We term this model as Gaussian Wiretap channel with Jammer-Aided eavesdropper (Gaussian WT-JA in short form). Note that this model recovers as a special case the Gaussian wiretap channel [2].

### 2.4. Special Case 2: The Gaussian Multiple-Access Wiretap Channel in the Presence of a Jammer-Aided Eavesdropper

**Figure 2:**
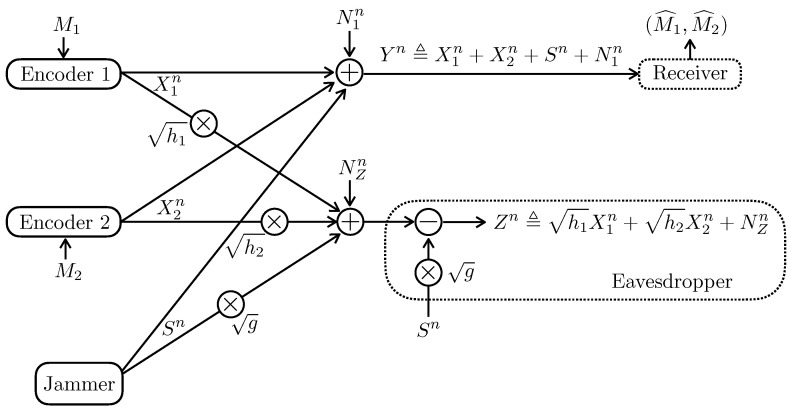
The Gaussian multiple-access wiretap channel in the presence of a jammer-aided eavesdropper.

Assume that the two receivers are colocated in Section 2.2. More specifically, as depicted in Figure 2, the channel model of Section 2.2 becomes

(4a) $$\begin{array}{cc} Y^{n} & ≜X_{1}^{n}+X_{2}^{n}+S^{n}+N_{1}^{n}, \end{array}$$

(4b) $$\begin{array}{cc} Z^{n} & ≜\sqrt{h_{1}}X_{1}^{n}+\sqrt{h_{2}}X_{2}^{n}+N_{Z}^{n}, \end{array}$$

where $\sigma_{1}^{2}=\sigma_{Z}^{2}=1$. We term the model as Gaussian Multiple-Access Wiretap channel with Jammer-Aided eavesdropper (Gaussian MAC-WT-JA in short form) with the parameters $\left(\Gamma_{1},\Gamma_{2},h_{1},h_{2},\Lambda ,\sigma_{1}^{2},\sigma_{Z}^{2}\right)$. This model recovers as special cases the model in [24] in the absence of the security constraint (2b), and the Gaussian multiple-access wiretap channel [3]. Note that the model in [24] was introduced to study the presence of selfish transmitters via cooperative game theory, and that, similarly, the Gaussian MAC-WT-JA can be used to study the presence of selfish transmitters via coalitional game theory [25].

### 2.5. Special Case 3: The Gaussian Broadcast Wiretap Channel in the Presence of a Jammer-Aided Eavesdropper

**Figure 3:**
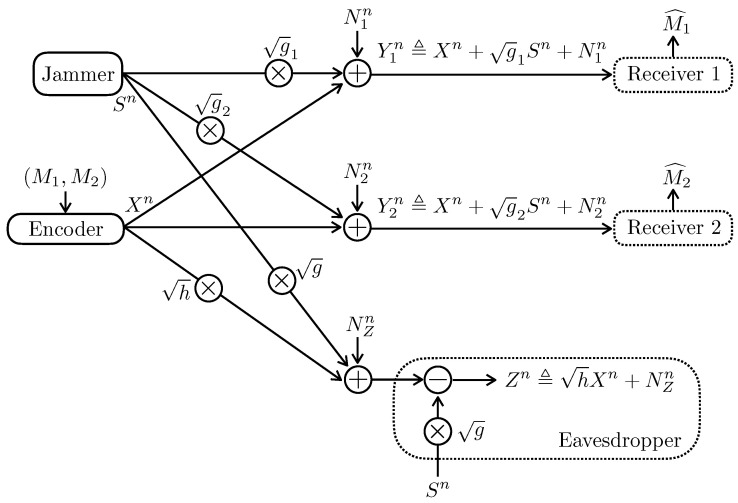
The Gaussian broadcast wiretap channel in the presence of a jammer-aided eavesdropper.

Assume that the two transmitters are colocated in Section 2.2. More specifically, as depicted in Figure 3, the channel model of Section 2.2 becomes

(5a) $$\begin{array}{cc} Y_{1}^{n} & ≜X^{n}+\sqrt{g_{1}}S^{n}+N_{1}^{n}, \end{array}$$

(5b) $$\begin{array}{cc} Y_{2}^{n} & ≜X^{n}+\sqrt{g_{2}}S^{n}+N_{2}^{n}, \end{array}$$

(5c) $$\begin{array}{cc} Z^{n} & ≜\sqrt{h}X^{n}+N_{Z}^{n}, \end{array}$$

where $\sigma_{Z}^{2}=1$. We term the model as Gaussian Broadcast Wiretap channel with Jammer-Aided eavesdropper (Gaussian BC-WT-JA in short form). Note that this model recovers as special cases the multi-receiver wiretap channel [26] and the model in [27] in the absence of the security constraint (2b).

### 2.6. Special Case 4: The Gaussian Symmetric Interference Wiretap Channel in the Presence of a Jammer-Aided Eavesdropper

Consider the following special case of the channel model of Section 2.2.

(6a) $$\begin{array}{cc} Y_{1}^{n} & ≜X_{1}^{n}+X_{2}^{n}+S^{n}+N_{1}^{n}, \end{array}$$

(6b) $$\begin{array}{cc} Y_{2}^{n} & ≜X_{1}^{n}+X_{2}^{n}+S^{n}+N_{2}^{n}, \end{array}$$

(6c) $$\begin{array}{cc} Z^{n} & ≜\sqrt{h_{1}}X_{1}^{n}+\sqrt{h_{2}}X_{2}^{n}+N_{Z}^{n}, \end{array}$$

where $\sigma_{1}^{2}=\sigma_{2}^{2}=\sigma_{Z}^{2}=1$. We term the model as Gaussian Symmetric Interference Wiretap channel with Jammer-Aided eavesdropper (Gaussian SI-WT-JA in short form). In the absence of the security constraint (2b) and the jamming sequence, this model recovers a special case of the Gaussian interference channel under strong interference [28].

## 3. The Gaussian Wiretap Channel in the Presence of a Jammer-Aided Eavesdropper

We present a capacity result for the Gaussian WT-JA model described in Section 2.3.

**Theorem** **1.** *The secrecy capacity of the Gaussian WT-JA is*

(7) $$\begin{array}{c} C\left(\Lambda \right)≜\left\{\begin{array}{cc} {\left[\frac{1}{2}\log\left(\frac{1+{\left(1+\Lambda \right)}^{-1}\Gamma }{1+h\Gamma }\right)\right]}^{+} & \;\mathrm{if}\;\Gamma >\Lambda \\ 0 & \;\mathrm{if}\;\Gamma \leq \Lambda \end{array}\right.. \end{array}$$

Observe that C(Λ) is non-zero if and only if Γ>Λ and ${\left(1+\Lambda \right)}^{-1}>h$. When Γ>Λ, Theorem 1 means that arbitrary oblivious jamming is no more harmful than Gaussian jamming, i.e., when the jamming sequence is obtained from independent and identical realizations of a zero-mean Gaussian random variable with variance equal to the power constraint Λ.

The proof of Theorem 1 follows as a special case of the achievability and converse bounds derived in the next section in Theorems 2 and 3, respectively, for the Gaussian MAC-WT-JA.

## 4. The Gaussian Multiple-Access Wiretap Channel in the Presence of a Jammer-Aided Eavesdropper

### 4.1. Inner and Outer Bounds for the Gaussian MAC-WT-JA

We derive inner and outer bounds for the Gaussian MAC-WT-JA in Theorems 2 and 3. Their proofs are provided in Section 7 and Section 8, respectively.

**Theorem** **2** (Achievability). *We consider three cases.*

1. *When $\Gamma_{1}>\Lambda$ and $\Gamma_{2}\leq \Lambda$,*
(8) $$\begin{array}{cc} \; & R_{1}^{MAC}≜\left\{\left(R_{1},0\right):R_{1}\leq \max_{0\leq P_{2}\leq \Gamma_{2}}{\left[\frac{1}{2}\log\left(\frac{1+\Gamma_{1}{\left(1+\Lambda +P_{2}\right)}^{-1}}{1+\Gamma_{1}h_{1}{\left(1+h_{2}P_{2}\right)}^{-1}}\right)\right]}^{+}\right\} \end{array}$$
  *is achievable.*
2. *When $\Gamma_{2}>\Lambda$ and $\Gamma_{1}\leq \Lambda$,*
(9) $$\begin{array}{cc} \; & R_{2}^{MAC}≜\left\{\left(0,R_{2}\right):R_{2}\leq \max_{0\leq P_{1}\leq \Gamma_{1}}{\left[\frac{1}{2}\log\left(\frac{1+\Gamma_{2}{\left(1+\Lambda +P_{1}\right)}^{-1}}{1+\Gamma_{2}h_{2}{\left(1+h_{1}P_{1}\right)}^{-1}}\right)\right]}^{+}\right\} \end{array}$$
  *is achievable.*
3. *When $\min(\Gamma_{1},\Gamma_{2})>\Lambda$,*
(10) $$\begin{array}{c} R^{MAC}≜Conv\left(R_{1}^{MAC}\cup R_{2}^{MAC}\cup \;\;\;\underset{\begin{array}{c} \Lambda <P_{1}\leq \Gamma_{1}\\ \Lambda <P_{2}\leq \Gamma_{2} \end{array}}{⋃}\;\;\;R_{1,2}^{MAC}\left(P_{1},P_{2}\right)\right) \end{array}$$
  *is achievable, where*
(11) R1,2MAC(P1,P2)≜∑TΛ(R1,R2):R1≤12log1+P1(1+Λ)−11+P1h1(1+h2P2)−1+,R2≤12log1+P2(1+Λ)−11+P2h2(1+h1P1)−1+,R1+R2≤12log1+(P1+P2)(1+Λ)−11+P1h1+P2h2+.

**Theorem** **3** (Partial Converse).

1. *If $\max(\Gamma_{1},\Gamma_{2})\leq \Lambda$, then no positive rate is achievable.*
2. *When $\min(\Gamma_{1},\Gamma_{2})>\Lambda$ and $h_{1}=h_{2}$, the sum-rate bound of $R_{1,2}^{MAC}\left(\Gamma_{1},\Gamma_{2}\right)$ described in Equation (11) is tight by choosing $\left(P_{1},P_{2}\right)=\left(\Gamma_{1},\Gamma_{2}\right)$.*

Observe that in the achievability scheme for $R_{1}^{MAC}$, choosing a transmission power smaller than $\Gamma_{1}$ for Transmitter 1 would result in a smaller region, since for a fixed $P_{2}$, $x\mapsto \log\left(\frac{1+x{\left(1+\Lambda +P_{2}\right)}^{-1}}{1+xh_{1}{\left(1+h_{2}P_{2}\right)}^{-1}}\right)$ is either negative when ${\left(1+\Lambda +P_{2}\right)}^{-1}\leq h_{1}{\left(1+h_{2}P_{2}\right)}^{-1}$, or non-decreasing when ${\left(1+\Lambda +P_{2}\right)}^{-1}>h_{1}{\left(1+h_{2}P_{2}\right)}^{-1}$. By exchanging the role of the transmitters, we have the same observation for $R_{2}^{MAC}$.

### 4.2. Discussion of Rate-Splitting

Rate-splitting [5] can be adapted to the Gaussian MAC-WT-JA to avoid time-sharing, however, the entire region in Equation (11) cannot be achieved as splitting the power of one user precludes reliable communication. Assuming that

(12) $$\begin{array}{c} I\left(X_{1}X_{2};Y\right)-I\left(X_{1}X_{2};Z\right)\geq \max\left[I\left(X_{1};Y|X_{2}\right)-I\left(X_{1};Z\right),I\left(X_{2};Y|X_{1}\right)-I\left(X_{2};Z\right)\right], \end{array}$$

then one can split the power of Transmitter 1 in $\left(P_{1}-\delta \right)$ and δ, where $\delta \in [0,P_{1}]$, and define the following functions from $\left[0,P_{1}\right]$ to R

(13a) $$\begin{array}{cc} R_{U}: & \delta \mapsto \frac{1}{2}\log\frac{1+\left(P_{1}-\delta \right){\left(1+\Lambda +\delta +P_{2}\right)}^{-1}}{1+h_{1}\left(P_{1}-\delta \right)}, \end{array}$$

(13b) $$\begin{array}{cc} R_{V}: & \delta \mapsto \frac{1}{2}\log\frac{1+\delta {\left(1+\Lambda \right)}^{-1}}{1+h_{1}\delta {\left(1+h_{1}\left(P_{1}-\delta \right)+h_{2}P_{2}\right)}^{-1}}, \end{array}$$

(13c) $$\begin{array}{cc} R_{2}: & \delta \mapsto \frac{1}{2}\log\frac{1+P_{2}{\left(1+\Lambda +\delta \right)}^{-1}}{1+h_{2}P_{2}{\left(1+h_{1}\left(P_{1}-\delta \right)\right)}^{-1}}. \end{array}$$

**Lemma** **1.** *For any $\delta \in [0,P_{1}]$, we have $\left(R_{U}+R_{V}+R_{2}\right)\left(\delta \right)=I\left(X_{1}X_{2};Y\right)-I\left(X_{1}X_{2};Z\right)$. Moreover, for any point $\left(x_{0},y_{0}\right)$ in*

(14) $$\begin{array}{cc} \; & D(P_{1},P_{2})\\ \; & ≜\left\{\left(R_{1},R_{2}\right)\in R_{1,2}^{MAC}\left(P_{1},P_{2}\right):R_{1}+R_{2}={\left[\frac{1}{2}\log\left(\frac{1+\left(P_{1}+P_{2}\right){\left(1+\Lambda \right)}^{-1}}{1+P_{1}h_{1}+P_{2}h_{2}}\right)\right]}^{+}\right\}, \end{array}$$

*there exists $\delta_{0}\in \left[0,P_{1}\right]$ such that $x_{0}=\left(R_{U}+R_{V}\right)\left(\delta_{0}\right)$ and $y_{0}=R_{2}\left(\delta_{0}\right)$.*

**Proof.**  Define

(15a) $$\begin{array}{cc} Y & ≜U+V+X_{2}+N_{Y}, \end{array}$$

(15b) $$\begin{array}{cc} Z & ≜\sqrt{h_{1}}\left(U+V\right)+\sqrt{h_{2}}X_{2}+N_{Z}, \end{array}$$

where *V*, *U*, $X_{2}$, $N_{Y}$, $N_{Z}$ are independent zero-mean Gaussian random variables with variances $\delta \in [0,P_{1}]$, $P_{1}-\delta$, $P_{2}$, (1+Λ), 1, respectively. Additionally, define

(16a) $$\begin{array}{cc} R_{U}\left(\delta \right) & ≜I\left(U;Y\right)-I\left(U;Z|VX_{2}\right)=\frac{1}{2}\log\frac{1+\left(P_{1}-\delta \right){\left(1+\Lambda +\delta +P_{2}\right)}^{-1}}{1+h_{1}\left(P_{1}-\delta \right)}, \end{array}$$

(16b) $$\begin{array}{cc} R_{V}\left(\delta \right) & ≜I\left(V;Y|UX_{2}\right)-I\left(V;Z\right)=\frac{1}{2}\log\frac{1+\delta {\left(1+\Lambda \right)}^{-1}}{1+h_{1}\delta {\left(1+h_{1}\left(P_{1}-\delta \right)+h_{2}P_{2}\right)}^{-1}}, \end{array}$$

(16c) $$\begin{array}{cc} R_{2}\left(\delta \right) & ≜I\left(X_{2};Y|U\right)-I\left(X_{2};Z|V\right)=\frac{1}{2}\log\frac{1+P_{2}{\left(1+\Lambda +\delta \right)}^{-1}}{1+h_{2}P_{2}{\left(1+h_{1}\left(P_{1}-\delta \right)\right)}^{-1}}. \end{array}$$

By the chain rule, we have, for any $\delta \in [0,P_{1}]$, $\left(R_{U}+R_{V}+R_{2}\right)\left(\delta \right)=I\left(X_{1}X_{2};Y\right)-I\left(X_{1}X_{2};Z\right)$. Finally, since $\left(R_{U}+R_{V}\right)\left(0\right)=I\left(X_{1};Y\right)-I\left(X_{1};Z|X_{2}\right)$ and $\left(R_{U}+R_{V}\right)\left(P_{1}\right)=I\left(X_{1};Y|X_{2}\right)-I\left(X_{1};Z\right)$, by continuity of $\delta \mapsto \left(R_{U}+R_{V}\right)\left(\delta \right)$, there exists $\delta_{0}\in \left[0,P_{1}\right]$ such that $x_{0}=\left(R_{U}+R_{V}\right)\left(\delta_{0}\right)$ and $y_{0}=R_{2}\left(\delta_{0}\right)$ for any point $\left(x_{0},y_{0}\right)$ in $D(P_{1},P_{2})$. □

As remarked in [29], a potential issue is that $R_{U}\left(\delta_{0}\right)$ or $R_{V}\left(\delta_{0}\right)$ can be negative in Lemma 1. We have the following achievability result.

**Proposition** **1.** *Let $\left(x_{0},y_{0}\right)\in D\left(P_{1},P_{2}\right)$ and $\delta_{0}$ be as in Lemma 1. Then, $\left(x_{0},y_{0}\right)$ can be achieved without time-sharing if $R_{U}\left(\delta_{0}\right)\geq 0$ and $R_{V}\left(\delta_{0}\right)\geq 0$ and $\min(\delta_{0},P_{1}-\delta_{0})>\Lambda$. $\left(x_{0},y_{0}\right)\in D\left(P_{1},P_{2}\right)$ can also be achieved without time-sharing if similar conditions (obtained by exchanging the role of the two transmitters) are satisfied when splitting the power of Transmitter 2.*

**Proof** **idea.** Transmitter 1 is split into two *virtual* users that transmit at rate $R_{U}\left(\delta \right)$ with power δ and at rate $R_{V}\left(\delta \right)$ with power $P_{1}-\delta$. Encoding for User 2 and the two virtual users is similar to Case 1 in the proof of Theorem 2. The receiver adopts a minimum distance decoding rule as in Theorem 2 to first decode the message associated with the virtual user that transmits at rate $R_{V}$, then to decode the message associated with User 2, and finally, to decode the message associated with the virtual user that transmits at rate $R_{U}$. A similar procedure can be performed if one decides to split the power of Transmitter 2. □

**Figure 4:**
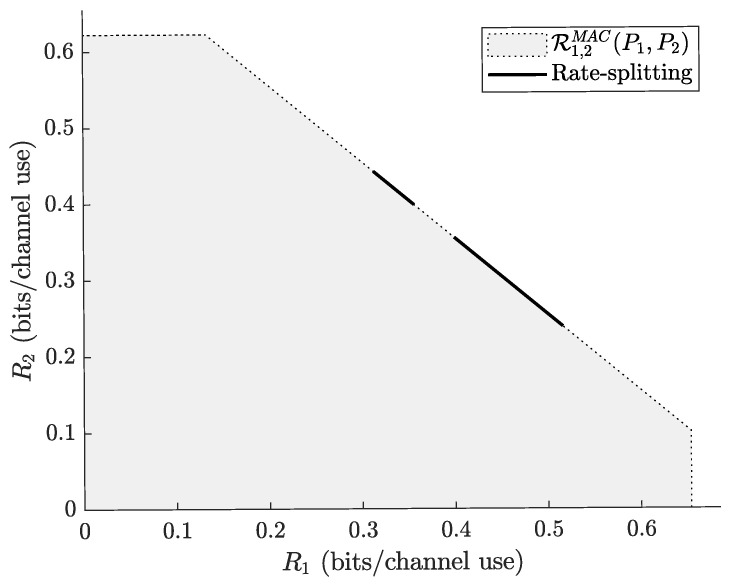
The shaded area represents $R_{1,2}^{MAC}\left(P_{1},P_{2}\right)$, where $\left(P_{1},P_{2},\Lambda ,h_{1},h_{2}\right)=\left(4,3.3,1.5,0.12,0.11\right)$. The solid segments represent the rate pairs achievable with Proposition 1.

An illustration of Proposition 1 is depicted in Figure 4. Note that for some model parameters, the set of points achievable with Proposition 1 can be empty and, unfortunately, it does not seem easy to obtain a simple analytical characterization of the rate pairs achievable with Proposition 1.

### 4.3. Extension to More Than Two Transmitters

We extend our result for the MAC-WT-JA to the case of an arbitrary number of transmitters. The problem is more involved than the case of two transmitters and requires new time-sharing strategies that leverage extended polymatroid properties.

Consider the model of Section 2.4 with *L* transmitters instead of two transmitters. We let L≜⟦1,L⟧ denote the set of transmitters. More specifically, the channel model of Section 2.4 becomes

(17a) $$\begin{array}{cc} Y^{n} & ≜\sum_{l\in L}X_{l}^{n}+S^{n}+N_{1}^{n}, \end{array}$$

(17b) $$\begin{array}{cc} Z^{n} & ≜\sum_{l\in L}\sqrt{h_{l}}X_{l}^{n}+N_{Z}^{n}, \end{array}$$

where $\sigma_{1}^{2}=\sigma_{Z}^{2}=1$. We term the model as Gaussian MAC-WT-JA with parameters $\left({\left(\Gamma_{l}\right)}_{l\in L},{\left(h_{l}\right)}_{l\in L},\Lambda ,\sigma_{1}^{2},\sigma_{Z}^{2}\right)$. When the channel gains ${\left(h_{l}\right)}_{l\in L}$ are all equal to h∈[0,1[, we refer to this model as the degraded MAC-WT-JA with parameters $\left({\left(\Gamma_{l}\right)}_{l\in L},h,\Lambda ,\sigma_{1}^{2},\sigma_{Z}^{2}\right)$. Given $\Lambda \in R_{+}$ and ${\left(\Gamma_{l}\right)}_{l\in L}$, we define $h_{\Lambda }≜{\left(1+\Lambda \right)}^{-1}$, $L\left(\Lambda \right)≜\left\{l\in L:\Gamma_{l}>\Lambda \right\}$, and $L^{c}\left(\Lambda \right)≜L\setminus L(\Lambda ).$ The following achievability result is proven in Appendix B.

**Theorem** **4.** *Assume that for all l∈L(Λ), $h_{\Lambda }>h_{l}$. The following region is achievable for the Gaussian MAC-WT-JA with parameters $\left({\left(\Gamma_{l}\right)}_{l\in L},{\left(h_{l}\right)}_{l\in L},\Lambda ,1,1\right)$*

(18) R=⋃(Pl)l∈L:∀l∈L(Λ),Λ<Pl≤Γl∑TΛ(Rl)l∈L:∀l∈Lc(Λ),Rl=0and∀T⊆L(Λ),−−−−−−−−−−−RT≤12log1+hΛPT1+(∑l∈ThlPl)(1+∑l∈TchlPl)−1+,

*where for any ${\left(P_{l}\right)}_{l\in L}$ and T⊆L, we use the notation $P_{T}≜\sum_{l\in T}P_{l}$.*

We immediately obtain the following corollary.

**Corollary** **1.** *The following region is achievable for the degraded Gaussian MAC-WT-JA with parameters $\left({\left(\Gamma_{l}\right)}_{l\in L},h,\Lambda ,1,1\right)$*

(19) R=⋃(Pl)l∈L:∀l∈L(Λ),Λ<Pl≤Γl∑TΛ(Rl)l∈L:∀l∈Lc(Λ),Rl=0and∀T⊆L(Λ),−−−−−−−−−−−RT≤12log1+hΛPT1+hPT(1+hPTc)−1+.

Note that the achievability strategy used in the proof of Theorem 4 is different than the achievability strategy used in the proof of Theorem 2. While Theorem 4 gains in generality by considering an arbitrary number of users, it requires the assumption $\forall l\in L\left(\Lambda \right),h_{\Lambda }>h_{l}$, which is not needed in Theorem 2. We also have the following optimality result, which is proven in Appendix C.

**Theorem** **5.** *The maximal secrecy sum-rate $R_{L}≜\sum_{l\in L}R_{l}$ achievable for the degraded Gaussian MAC-WT-JA with parameters $\left({\left(\Gamma_{l}\right)}_{l\in L},h,\Lambda ,1,1\right)$ is*

(20) $$\begin{array}{c} {\left[\frac{1}{2}\log\left(\frac{1+h_{\Lambda }\Gamma_{L(\Lambda )}}{1+h\Gamma_{L(\Lambda )}}\right)\right]}^{+}. \end{array}$$

Note that the optimal secrecy sum-rate is positive if and only if $h_{\Lambda }>h$ and L(Λ)≠∅.

## 5. The Gaussian Broadcast Wiretap Channel in the Presence of a Jammer-Aided Eavesdropper

Theorems 6 and 7 provide inner and outer bounds, respectively, for the Gaussian BC-WT-JA.

**Theorem** **6** (Achievability). *We have the following inner bounds.*

1. *When $g_{2}\Lambda \geq \Gamma$ and $g_{1}\Lambda <\Gamma$,*
(21) $$\begin{array}{c} R_{1}^{BC}≜\left\{\left(R_{1},0\right):R_{1}\leq {\left[\frac{1}{2}\log\left(\frac{1+\frac{\Gamma }{\sigma_{1}^{2}+g_{1}\Lambda }}{1+h\Gamma }\right)\right]}^{+}\right\} \end{array}$$
  *is achievable.*
2. *When $g_{1}\Lambda \geq \Gamma$ and $g_{2}\Lambda <\Gamma$,*
(22) $$\begin{array}{c} R_{2}^{BC}≜\left\{\left(0,R_{2}\right):R_{2}\leq {\left[\frac{1}{2}\log\left(\frac{1+\frac{\Gamma }{\sigma_{2}^{2}+g_{2}\Lambda }}{1+h\Gamma }\right)\right]}^{+}\right\} \end{array}$$
  *is achievable.*
3. *When $\max(g_{1}\Lambda ,g_{2}\Lambda )<\Gamma$, and, without loss of generality, $\sigma_{1}^{2}+g_{1}\Lambda \leq \sigma_{2}^{2}+g_{2}\Lambda$ (exchange the role of the receivers if $\sigma_{1}^{2}+g_{1}\Lambda >\sigma_{2}^{2}+g_{2}\Lambda$),*
(23) $$\begin{array}{c} \mathrm{Conv}\left(R_{1}^{BC}\cup R_{2}^{BC}\cup \underset{\alpha \in ]\max\left(g_{1},g_{2}\right)\Lambda \Gamma^{-1},1]}{⋃}R^{BC}\left(\alpha \right)\right), \end{array}$$
  *is achievable where we have defined for α∈[0,1]*
(24) RBC(α)≜(R1,R2):R1≤12log1+(1−α)Γσ12+g1Λ1+h(1−α)Γ+,.−−−−−−R2≤12log1+αΓ(1−α)Γ+σ22+g2Λ1+hαΓh(1−α)Γ+1+.

Note that $R^{BC}\left(\alpha =0\right)=R_{1}^{BC}$ and $R^{BC}\left(\alpha =1\right)=R_{2}^{BC}$. The achievability scheme of Theorem 6 is similar to the proof of Theorem 2 and [27] [Theorem 3].

**Theorem** **7** (Partial converse).

1. *If $\Gamma \leq \min(g_{1}\Lambda ,g_{2}\Lambda )$, then no positive rate is achievable;*
2. *When $g_{2}\Lambda \geq \Gamma$ and $g_{1}\Lambda <\Gamma$, the achievability region $R_{1}^{BC}$ in Theorem 6 is tight;*
3. *When $g_{1}\Lambda \geq \Gamma$ and $g_{2}\Lambda <\Gamma$, the achievability region $R_{2}^{BC}$ in Theorem 6 is tight;*
4. *When $\Gamma >\max(g_{1}\Lambda ,g_{2}\Lambda )$, the following region is an outer bound*
(25) $$\begin{array}{c} \underset{\alpha \in [0,1]}{⋃}R^{BC}\left(\alpha \right), \end{array}$$
  *where $R^{BC}\left(\alpha \right)$ has been defined in Theorem 6.*

The proof of Theorem 7 is similar to the proof of Theorem 3 using [26] in place of [30]. Observe that the gap between the inner and outer bounds of Theorems 6 and 7 when $\Gamma >\max(g_{1}\Lambda ,g_{2}\Lambda )$ comes from the fact that our achievability scheme is limited to $\alpha \in ]\max\left(g_{1},g_{2}\right)\Lambda \Gamma^{-1},1]\cup \left\{0\right\}$.

## 6. The Symmetric Interference Wiretap Channel in the Presence of a Jammer-Aided Eavesdropper

By the symmetry in Equation (6a) and Equation (6b), a code for the Gaussian MAC-WT-JA allows Receiver i∈{1,2} to securely recover the message of Transmitter *i*. Hence, from the achievability result for the Gaussian MAC-WT-JA, we have the following achievability result for the Gaussian SI-WT-JA.

**Theorem** **8** (Achievability). *We consider three cases.*

1. *When $\Gamma_{1}>\Lambda$ and $\Gamma_{2}\leq \Lambda$, $R_{1}^{SI}≜R_{1}^{MAC}$ is achievable;*
2. *When $\Gamma_{2}>\Lambda$ and $\Gamma_{1}\leq \Lambda$, $R_{2}^{SI}≜R_{2}^{MAC}$ is achievable;*
3. *When $\min(\Gamma_{1},\Gamma_{2})>\Lambda$, $R^{SI}≜R^{MAC}$ is achievable;*

*where $R_{1}^{MAC}$, $R_{2}^{MAC}$, and $R^{MAC}$ are defined in Theorem 2.*

Next, by the symmetry in Equations (6a) and (6b), we have that any code for the Gaussian SI-WT-JA allows Receiver i∈{1,2} to securely recover the messages from both transmitters, meaning that an outer bound for the Gaussian SI-WT-JA can be obtained by considering an outer bound for a Gaussian MAC-WT-JA. Hence, from the partial converse for the Gaussian MAC-WT-JA, we obtain the following partial converse for the Gaussian SI-WT-JA.

**Theorem** **9** (Partial converse).

1. *If $\max(\Gamma_{1},\Gamma_{2})\leq \Lambda$, then no positive rate is achievable.*
2. *When $\min(\Gamma_{1},\Gamma_{2})>\Lambda$ and $h_{1}=h_{2}$, the sum-rate achieved in $R^{SI}$ is tight by choosing $\left(P_{1},P_{2}\right)=\left(\Gamma_{1},\Gamma_{2}\right)$.*

## 7. Proof of Theorem 2

**Figure 5:**
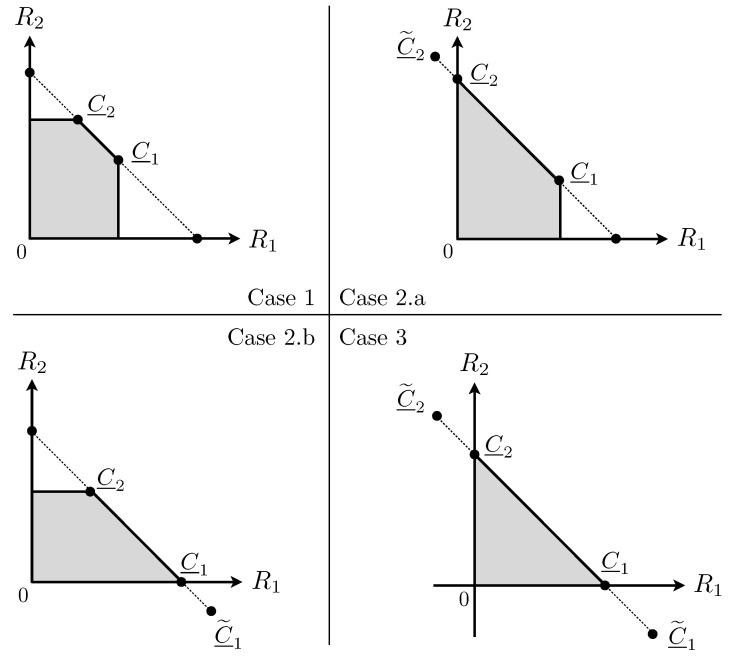
Region $R_{1,2}\left(P_{1},P_{2}\right)$.

To prove Theorem 2, it is sufficient to prove the achievability of the dominant face

(26) $$\begin{array}{cc} \; & D(P_{1},P_{2})\\ \; & ≜\left\{\left(R_{1},R_{2}\right)\in R_{1,2}^{MAC}\left(P_{1},P_{2}\right):R_{1}+R_{2}={\left[\frac{1}{2}\log\left(\frac{1+\left(P_{1}+P_{2}\right){\left(1+\Lambda \right)}^{-1}}{1+P_{1}h_{1}+P_{2}h_{2}}\right)\right]}^{+}\right\} \end{array}$$

of $R_{1,2}^{MAC}\left(P_{1},P_{2}\right)$ to prove the achievability of $R_{1,2}^{MAC}\left(P_{1},P_{2}\right)$ when $\min(\Gamma_{1},\Gamma_{2})>\Lambda$ and where $\left(P_{1},P_{2}\right)\in ]\Lambda ,\Gamma_{1}]\times ]\Lambda ,\Gamma_{2}]$. The achievability of $R_{i}^{MAC}$, i∈{1,2}, when $\Gamma_{i}>\Lambda$ and $\Gamma_{3-i}\leq \Lambda$ is obtained similarly by having Transmitter $\bar{i}≜3-i$ send Gaussian noise. Observe that the rate constraints in $R_{1,2}^{MAC}\left(P_{1},P_{2}\right)$ can be expressed as

(27a) $$\begin{array}{cc} R_{1} & \leq {\left[I\left(X_{1};Y|X_{2}\right)-I\left(X_{1};Z\right)\right]}^{+}, \end{array}$$

(27b) $$\begin{array}{cc} R_{2} & \leq {\left[I\left(X_{2};Y|X_{1}\right)-I\left(X_{2};Z\right)\right]}^{+}, \end{array}$$

(27c) $$\begin{array}{cc} R_{1}+R_{2} & \leq {\left[I\left(X_{1}X_{2};Y\right)-I\left(X_{1}X_{2};Z\right)\right]}^{+}, \end{array}$$

where

(28a) $$\begin{array}{cc} Y & ≜X_{1}+X_{2}+N_{Y}, \end{array}$$

(28b) $$\begin{array}{cc} Z & ≜\sqrt{h_{1}}X_{1}+\sqrt{h_{2}}X_{2}+N_{Z}, \end{array}$$

and $X_{1}$, $X_{2}$, $N_{Y}$, $N_{Z}$ are independent zero-mean Gaussian random variables with variances $P_{1}$, $P_{2}$, (1+Λ), 1, respectively. As remarked in [29], the set function $T\mapsto I\left(X_{T};Y|X_{T^{c}}\right)-I\left(X_{T};Z\right)$ is submodular but not necessarily non-decreasing, where ∀T⊆{1,2}, $X_{T}≜{\left(X_{t}\right)}_{t\in T}$. This is the main reason why achieving the corner points of $R_{1,2}^{MAC}\left(P_{1},P_{2}\right)$ by means of point-to-point codes via the successive decoding method [5] [Appendix C] does not easily translate to our setting. Before we provide our solution, we summarize our proof strategy in the three cases below. Figure 5 illustrates these cases.

**Case 1**: Assume

(29) $$\begin{array}{c} I\left(X_{1}X_{2};Y\right)-I\left(X_{1}X_{2};Z\right)\geq \max\left[I\left(X_{1};Y|X_{2}\right)-I\left(X_{1};Z\right),I\left(X_{2};Y|X_{1}\right)-I\left(X_{2};Z\right)\right]. \end{array}$$

The corner points of $R_{1,2}^{MAC}$ are given by

(30a) $$\begin{array}{cc} {\underline{C}}_{1} & ≜(I\left(X_{1};Y|X_{2}\right)-I\left(X_{1};Z\right),I\left(X_{2};Y\right)-I\left(X_{2};Z|X_{1}\right)), \end{array}$$

(30b) $$\begin{array}{cc} {\underline{C}}_{2} & ≜(I\left(X_{1};Y\right)-I\left(X_{1};Z|X_{2}\right),I\left(X_{2};Y|X_{1}\right)-I\left(X_{2};Z\right)). \end{array}$$

We will achieve each corner point with point-to-point coding techniques and perform time-sharing to achieve $D(P_{1},P_{2})$. Specifically, to achieve ${\underline{C}}_{i}$, i∈{1,2}, the encoders will be designed such that the decoder can first estimate the codeword sent by Transmitter $\bar{i}≜3-i$ (by considering the codewords of Transmitter *i* as noise), which is in turn used to estimate the codeword sent by Transmitter *i*. This approach is similar to the successive decoding method [5] [Appendix C] for a multiple-access channel in the absence of a security constraint.

**Case 2.a**: Assume

(31a) $$\begin{array}{cc} I\left(X_{1}X_{2};Y\right)-I\left(X_{1}X_{2};Z\right) & \geq I\left(X_{1};Y|X_{2}\right)-I\left(X_{1};Z\right), \end{array}$$

(31b) $$\begin{array}{cc} I\left(X_{1}X_{2};Y\right)-I\left(X_{1}X_{2};Z\right) & <I\left(X_{2};Y|X_{1}\right)-I\left(X_{2};Z\right). \end{array}$$

Hence,

(32) $$\begin{array}{c} {\tilde{\underline{C}}}_{2}≜\left(I\left(X_{1};Y\right)-I\left(X_{1};Z|X_{2}\right),I\left(X_{2};Y|X_{1}\right)-I\left(X_{2};Z\right)\right) \end{array}$$

has a negative x-coordinate and the method of Case 1 cannot be directly applied here. Now, the corner points of $R_{1,2}^{MAC}$ are

(33a) $$\begin{array}{cc} {\underline{C}}_{1} & ≜(I\left(X_{1};Y|X_{2}\right)-I\left(X_{1};Z\right),I\left(X_{2};Y\right)-I\left(X_{2};Z|X_{1}\right)), \end{array}$$

(33b) $$\begin{array}{cc} {\underline{C}}_{2} & ≜(0,I\left(X_{1}X_{2};Y\right)-I\left(X_{1}X_{2};Z\right))). \end{array}$$

The idea to achieve ${\underline{C}}_{1}$ is, as in Case 1, a successive decoding approach by decomposing the sum rate $I\left(X_{1}X_{2};Y\right)-I\left(X_{1}X_{2};Z\right)$ as the sum of $I\left(X_{2};Y\right)-I\left(X_{2};Z|X_{1}\right)$, which represents the secret message rate for Transmitter 2, and $I\left(X_{1};Y|X_{2}\right)-I\left(X_{1};Z\right)$, which represents the secret message rate for Transmitter 1. However, ${\underline{C}}_{2}$ cannot be decomposed in a similar manner and thus cannot be achieved with the same method. Instead, to achieve any point in $D(P_{1},P_{2})$, we rely on a strategy over several transmission blocks. First, in an appropriate number of transmission blocks, the transmitters can send secret messages with rates ${\underline{C}}_{1}$ as in Case 1. Part of the secret messages of Transmitter 1, with a rate equal to the absolute value of the x-coordinate of the point ${\tilde{\underline{C}}}_{2}$, is dedicated to the exchange of a secret key between Transmitter 1 and the legitimate receiver. Then, for the remaining transmission blocks, Transmitter 2 transmits a secret message with rate $I\left(X_{1}X_{2};Y\right)-I\left(X_{1}X_{2};Z\right)$, while Transmitter 1 uses the previously generated secret key to produce a jamming signal, which can be canceled out by the legitimate receiver but not by the eavesdropper who does not know the secret key.

**Case 2.b**: Assume

(34a) $$\begin{array}{cc} I\left(X_{1}X_{2};Y\right)-I\left(X_{1}X_{2};Z\right) & \geq I\left(X_{2};Y|X_{1}\right)-I\left(X_{2};Z\right), \end{array}$$

(34b) $$\begin{array}{cc} I\left(X_{1}X_{2};Y\right)-I\left(X_{1}X_{2};Z\right) & <I\left(X_{1};Y|X_{2}\right)-I\left(X_{1};Z\right). \end{array}$$

This case is handled as Case 2.a by exchanging the role of the two transmitters.

**Case 3**: Assume

(35) $$\begin{array}{c} I\left(X_{1}X_{2};Y\right)-I\left(X_{1}X_{2};Z\right)<\min\left[I\left(X_{1};Y|X_{2}\right)-I\left(X_{1};Z\right),I\left(X_{2};Y|X_{1}\right)-I\left(X_{2};Z\right)\right]. \end{array}$$

Hence,

(36a) $$\begin{array}{cc} {\tilde{\underline{C}}}_{1} & ≜(I\left(X_{1};Y|X_{2}\right)-I\left(X_{1};Z\right),I\left(X_{2};Y\right)-I\left(X_{2};Z|X_{1}\right)), \end{array}$$

(36b) $$\begin{array}{cc} {\tilde{\underline{C}}}_{2} & ≜(I\left(X_{1};Y\right)-I\left(X_{1};Z|X_{2}\right),I\left(X_{2};Y|X_{1}\right)-I\left(X_{2};Z\right)), \end{array}$$

have a negative y-component and a negative x-component, respectively, and the strategy of Case 1 or Case 2 cannot be directly applied here. The corner points of the region are

(37a) $$\begin{array}{cc} {\underline{C}}_{1} & ≜(I\left(X_{1}X_{2};Y\right)-I\left(X_{1}X_{2};Z\right),0), \end{array}$$

(37b) $$\begin{array}{cc} {\underline{C}}_{2} & ≜(0,I\left(X_{1}X_{2};Y\right)-I\left(X_{1}X_{2};Z\right)). \end{array}$$

These corner points do not seem to be easily achievable using the method for Case 1. We will first show that it is possible to achieve a point $\underline{R}\in D\left(P_{1},P_{2}\right)$, where $\underline{R}$ has strictly positive components. All the other points in $D(P_{1},P_{2})$ will then be achieved as in Case 2 by doing the substitutions ${\underline{C}}_{1}\leftarrow \underline{R}$ and ${\underline{C}}_{2}\leftarrow \underline{R}$ in Case 2.a and Case 2.b, respectively.

Note that it is sufficient to consider the case

(38) $$\begin{array}{c} \min[I\left(X_{1};Y|X_{2}\right)-I\left(X_{1};Z\right),I\left(X_{2};Y|X_{1}\right)-I\left(X_{2};Z\right)]\geq 0. \end{array}$$

Indeed, for i∈{1,2} and $\bar{i}≜3-i$, when $I\left(X_{i};Y|X_{\bar{i}}\right)-I\left(X_{i};Z\right)>0$ and $I\left(X_{\bar{i}};Y|X_{i}\right)-I\left(X_{\bar{i}};Z\right)\leq 0$, we have $R_{\bar{i}}=0$ and $R_{i}\leq I\left(X_{1}X_{2};Y\right)-I\left(X_{1}X_{2};Z\right)\leq I\left(X_{i};Y|X_{\bar{i}}\right)-I\left(X_{i};Z|X_{\bar{i}}\right)=\frac{1}{2}\log\left(\frac{1+P_{i}{\left(1+\Lambda \right)}^{-1}}{1+P_{i}h_{i}}\right)$. These cases correspond to Theorem 1 and can be treated as in Case 1.

### 7.1. Case 1

We show the achievability of ${\underline{C}}_{2}$. The achievability of ${\underline{C}}_{1}$ is obtained by exchanging the role of the transmitters.

**Codebook construction**: For Transmitter i∈{1,2}, construct a codebook $C_{n}^{\left(i\right)}$ with $\left\lceil 2^{nR_{i}}\right\rceil \left\lceil 2^{n{\tilde{R}}_{i}}\right\rceil$ codewords drawn independently and uniformly on the sphere of radius $\sqrt{nP_{i}}$ in $R^{n}$. The codewords are labeled $x_{i}^{n}\left(m_{i},{\tilde{m}}_{i}\right)$, where $m_{i}\in ⟦1,2^{nR_{i}}⟧$, ${\tilde{m}}_{i}\in ⟦1,2^{n{\tilde{R}}_{i}}⟧$. We define $C_{n}≜\left(C_{n}^{\left(1\right)},C_{n}^{\left(2\right)}\right)$ and choose for δ>0

(39a) $$\begin{array}{cc} R_{1} & ≜I\left(X_{1};Y\right)-I\left(X_{1};Z|X_{2}\right)-\delta , \end{array}$$

(39b) $$\begin{array}{cc} {\tilde{R}}_{1} & ≜I(X_{1};Z|X_{2})-\delta , \end{array}$$

(39c) $$\begin{array}{cc} R_{2} & ≜I\left(X_{2};Y|X_{1}\right)-I\left(X_{2};Z\right)-\delta , \end{array}$$

(39d) $$\begin{array}{cc} {\tilde{R}}_{2} & ≜I(X_{2};Z)-\delta . \end{array}$$

**Encoding at Transmitter i∈{1,2}**: Given $\left(m_{i},{\tilde{m}}_{i}\right)$, transmit $x_{i}^{n}\left(m_{i},{\tilde{m}}_{i}\right)$. In the remainder of the paper, we use the term randomization sequence for ${\tilde{m}}_{i}$.

**Decoding**: The receiver performs minimum distance decoding to first estimate $\left(m_{1},{\tilde{m}}_{1}\right)$ and then to estimate $\left(m_{2},{\tilde{m}}_{2}\right)$, i.e., given $y^{n}$, it determines $\left({\hat{m}}_{1},{\hat{\tilde{m}}}_{1}\right)≜\phi_{1}\left(y^{n},0\right)$, and $\left({\hat{m}}_{2},{\hat{\tilde{m}}}_{2}\right)≜\phi_{2}\left(y^{n},x_{1}^{n}\left({\hat{m}}_{1},{\hat{\tilde{m}}}_{1}\right)\right)$ where for i∈{1,2}

(40) ϕi(yn,x)≜(mi,m˜i)if∥yn−x−xin(mi,m˜i)∥2<∥yn−x−xin(mi′,m˜i′)∥2−−−−−−−−−−−−for(mi′,m˜i′)≠(mi,m˜i)0ifnosuch(mi,m˜i)∈⟦1,2nRi⟧×⟦1,2nR˜i⟧exists.

Define $e\left(C_{n},s^{n}\right)≜P\left[\left({\hat{M}}_{1},{\hat{M}}_{2}\right)\neq \left(M_{1},M_{2}\right)|C_{n}\right]$. We now prove that $E_{C_{n}}\left[\sup_{s^{n}}e\left(C_{n},s^{n}\right)\right]$ $+\frac{1}{n}I\left(M_{1}M_{2};Z^{n}|C_{n}\right)\overset{n\to \infty }{\to }0$. We will thus conclude by Markov’s inequality that there exists a sequence of realizations ${\left(C_{n}\right)}_{n\geq 1}$ of ${\left(C_{n}\right)}_{n\geq 1}$ such that both $\sup_{s^{n}}e\left(C_{n},s^{n}\right)$ and $\frac{1}{n}I\left(M_{1}M_{2};Z^{n}|C_{n}\right)$ can be made arbitrarily close to zero as n→∞.

**Average probability of error**: We have

(41a) $$\begin{array}{cc} e(C_{n},s^{n})\; & \leq P\left[\left({\hat{M}}_{1},{\hat{M}}_{2}\right)\neq \left(M_{1},M_{2}\right)\;\mathrm{or}\;\left({\hat{\tilde{M}}}_{1},{\hat{\tilde{M}}}_{2}\right)\neq \left({\tilde{M}}_{1},{\tilde{M}}_{2}\right)|C_{n}\right] \end{array}$$

(41b) $$\begin{array}{cc} \; & \leq e_{1}\left(C_{n},s^{n},x_{2}^{n}\left(M_{2},{\tilde{M}}_{2}\right)\right)+e_{2}\left(C_{n},s^{n},0\right), \end{array}$$

where for i∈{1,2}

(42) ei(Cn,sn,x)≜1⌈2nRi⌉⌈2nR˜i⌉∑mi∑m˜iP∥xin(mi,m˜i)+sn+x+NYn−xin(mi′,m˜i′)∥2−−−−−−−−−≤∥sn+x+NYn∥2forsome(mi′,m˜i′)≠(mi,m˜i).

Next, we have

(43a) $$\begin{array}{cc} E_{C_{n}}\left[e_{1}\left(C_{n},s^{n},x_{2}^{n}\left(M_{2},{\tilde{M}}_{2}\right)\right)\right]\; & \leq E_{C_{n}}\left[e_{1}\left(C_{n},s^{n},x_{2}^{n}\left(M_{2},{\tilde{M}}_{2}\right)\right)|C_{n}^{\left(1\right)}\in C_{1}^{*}\right]+P\left[C_{n}^{\left(1\right)}\notin C_{1}^{*}\right] \end{array}$$

(43b) $$\begin{array}{cc} \; & \overset{n\to \infty }{\to }0, \end{array}$$

where, in Equation (43a), $C_{1}^{*}$ represents all the sets of unit norm vectors scaled by $\sqrt{nP_{1}}$ that satisfy the two conditions of Lemma A1 (in Appendix A), Equation (43b) holds because $P\left[C_{n}^{\left(1\right)}\in C_{1}^{*}\right]\overset{n\to \infty }{\to }1$ by Lemma A1, and $E_{C_{n}}\left[e_{1}\left(C_{n},s^{n},x_{2}^{n}\left(M_{2},{\tilde{M}}_{2}\right)\right)|C_{n}^{\left(1\right)}\in C_{1}^{*}\right]\overset{n\to \infty }{\to }0$ by Theorem A1 (in Appendix A) using that $R_{1}+{\tilde{R}}_{1}<I\left(X_{1};Y\right)=\frac{1}{2}\log\left(1+\frac{P_{1}}{1+\Lambda +P_{2}}\right)$ and by interpreting the signal of Transmitter 2 as noise. Then,

(44a) $$\begin{array}{cc} E_{C_{n}}\left[e_{2}\left(C_{n},s^{n},0\right)\right]\; & \leq E_{C_{n}}\left[e_{2}\left(C_{n},s^{n},0\right)|C_{n}^{\left(2\right)}\in C_{2}^{*}\right]+P\left[C_{n}^{\left(2\right)}\notin C_{2}^{*}\right] \end{array}$$

(44b) $$\begin{array}{cc} \; & \overset{n\to \infty }{\to }0, \end{array}$$

where, in Equation (44a), $C_{2}^{*}$ represents all the sets of unit norm vectors scaled by $\sqrt{nP_{2}}$ that satisfy the two conditions of Lemma A1, Equation (44b) holds because $P\left[C_{n}^{\left(2\right)}\in C_{2}^{*}\right]\overset{n\to \infty }{\to }1$ by Lemma A1, and $E_{C_{n}}\left[e_{2}\left(C_{n},s^{n},0\right)|C_{n}^{\left(2\right)}\in C_{2}^{*}\right]\overset{n\to \infty }{\to }0$ by Theorem A1 using that $R_{2}+{\tilde{R}}_{2}<I\left(X_{2};Y|X_{1}\right)=\frac{1}{2}\log\left(1+\frac{P_{2}}{1+\Lambda }\right)$. Hence, by Equations (41b), (43b) and (44b), we have

(45) $$\begin{array}{c} E_{C_{n}}\left[e\left(C_{n},s^{n}\right)\right]\overset{n\to \infty }{\to }0. \end{array}$$

**Equivocation**: We first study the average error probability of decoding $\left({\tilde{m}}_{1},{\tilde{m}}_{2}\right)$ given $\left(z^{n},m_{1},m_{2}\right)$ with the following procedure. Given $\left(z^{n},m_{1},m_{2}\right)$, determine ${\overset{˘}{m}}_{2}≜\psi_{2}\left(z^{n},0\right)$, and ${\overset{˘}{m}}_{1}≜\psi_{1}\left(z^{n},{\sqrt{h}}_{2}x_{2}^{n}\left(m_{2},{\overset{˘}{m}}_{2}\right)\right)$ where

(46) ψi(zn,x)≜m˜iif∥zn−x−hixin(mi,m˜i)∥2<∥zn−x−hixin(mi,m˜i′)∥2−−−−−−−−−−−−−−−−−−form˜i′≠m˜i0ifnosuchm˜i∈⟦1,2nR˜i⟧exists.

We define $\tilde{e}\left(C_{n}\right)≜P\left[\left({\overset{˘}{M}}_{1},{\overset{˘}{M}}_{2}\right)\neq \left({\tilde{M}}_{1},{\tilde{M}}_{2}\right)|C_{n}\right]$ and for i∈{1,2},

(47) e˜i(Cn,x)≜1⌈2nR˜i⌉∑m˜iP∥hixin(mi,m˜i)+x+NZn−hixin(mi,m˜i′)∥2−−−−−−−−−−−−≤∥x+NZn∥2forsomem˜i′≠m˜i.

Then, with the same notation as in Equations (43) and (44), we have

(48a) $$\begin{array}{cc} E_{C_{n}}\left[\tilde{e}\left(C_{n}\right)\right]\; & \leq E_{C_{n}}\left[{\tilde{e}}_{1}\left(C_{n},0\right)\right]+E_{C_{n}}\left[{\tilde{e}}_{2}\left(C_{n},{\sqrt{h}}_{1}x_{1}^{n}\left(M_{1},{\tilde{M}}_{1}\right)\right)\right]\\ \; & \leq E_{C_{n}}\left[{\tilde{e}}_{1}\left(C_{n},0\right)|C_{n}^{\left(1\right)}\in C_{1}^{*}\right]+P\left[C_{n}^{\left(1\right)}\notin C_{1}^{*}\right] \end{array}$$

(48b) −−                                        +ECn[e˜2(Cn,h1x1n(M1,M˜1))|Cn(2)∈C2*]+P[Cn(2)∉C2*]

(48c) $$\begin{array}{cc} \; & \overset{n\to \infty }{\to }0, \end{array}$$

where Equation (48c) holds because $P\left[C_{n}^{\left(1\right)}\in C_{1}^{*}\right]\overset{n\to \infty }{\to }1$ and $P\left[C_{n}^{\left(2\right)}\in C_{2}^{*}\right]\overset{n\to \infty }{\to }1$ by Lemma A1, $E_{C_{n}}\left[{\tilde{e}}_{1}\left(C_{n},0\right)|C_{n}^{\left(1\right)}\in C_{1}^{*}\right]\overset{n\to \infty }{\to }0$ by Theorem A1 using that ${\tilde{R}}_{1}<I\left(X_{1};Z|X_{2}\right)=\frac{1}{2}\log\left(1+h_{1}P_{1}\right)$, and $E_{C_{n}}\left[{\tilde{e}}_{2}\left(C_{n},\sqrt{h_{1}}x_{1}^{n}\left(M_{1},{\tilde{M}}_{1}\right)\right)|C_{n}^{\left(2\right)}\in C_{2}^{*}\right]\overset{n\to \infty }{\to }0$ by Theorem A1 using that ${\tilde{R}}_{2}<I\left(X_{2};Z\right)=\frac{1}{2}\log\left(1+\frac{h_{2}P_{2}}{1+h_{1}P_{1}}\right)$ and by interpreting the signal of Transmitter 1 as noise.

Define $M≜(M_{1},M_{2})$, $\tilde{M}≜\left({\tilde{M}}_{1},{\tilde{M}}_{2}\right)$. Let the superscript *T* denote the transpose operation and define $X≜{\left[\sqrt{h_{1}}{\left(X_{1}^{n}\right)}^{T}\;\sqrt{h_{2}}{\left(X_{2}^{n}\right)}^{T}\right]}^{T}\in R^{2n\times 1}$, such that

(49) $$\begin{array}{c} Z^{n}=GX+N_{Z}^{n}, \end{array}$$

with $G≜\left[I_{n},I_{n}\right]\in R^{n\times 2n}$ and $I_{n}$ the identity matrix with dimension *n*. Let $K_{X}$ denote the covariance matrix of X. Note that, by independence between $X_{1}^{n}$ and $X_{2}^{n}$, we have $K_{X}=\left(\begin{array}{cc} K_{\sqrt{h_{1}}X_{1}^{n}} & 0_{n}\\ 0_{n} & K_{\sqrt{h_{2}}X_{2}^{n}} \end{array}\right)$, where $0_{n}≜0\times I_{n}$ and $K_{\sqrt{h_{i}}X_{i}^{n}}$ is the covariance matrix of $\sqrt{h_{i}}X_{i}^{n}$, i∈{1,2}. Then, for i∈{1,2}, since $X_{i}^{n}$ is chosen uniformly at random over a sphere of radius $\sqrt{nP_{i}}$, the off-diagonal elements of $K_{\sqrt{h_{i}}X_{i}^{n}}$ are all equal to 0 by symmetry, and the diagonal elements are all equal (also by symmetry) and sum to $nh_{i}P_{i}$. Hence, $K_{\sqrt{h_{i}}X_{i}^{n}}=h_{i}P_{i}I_{n}$, i∈{1,2}, and

(50) $$\begin{array}{c} K_{X}=\left(\begin{array}{cc} h_{1}P_{1}I_{n} & 0_{n}\\ 0_{n} & h_{2}P_{2}I_{n} \end{array}\right). \end{array}$$

Then, we have

(51a) $$\begin{array}{cc} I(M;Z^{n}|C_{n})\; & =I\left(M\tilde{M};Z^{n}|C_{n}\right)-I\left(\tilde{M};Z^{n}|MC_{n}\right) \end{array}$$

(51b) $$\begin{array}{cc} \; & \;\;\;\;\;\;\;\;\;\;\;=I\left(M\tilde{M};Z^{n}|C_{n}\right)-H\left(\tilde{M}|C_{n}\right)+H\left(\tilde{M}|Z^{n}MC_{n}\right) \end{array}$$

(51c) $$\begin{array}{cc} \; & \;\;\;\;\;\;\;\;\;\;\;\;\;\;\;\;\;\;\;\;\;\;\;\;\;\;\;\;\;\;\;\;\;\;\;\;\;\;\leq I\left(X;Z^{n}|C_{n}\right)-H\left(\tilde{M}|C_{n}\right)+H\left(\tilde{M}|Z^{n}MC_{n}\right) \end{array}$$

(51d) $$\begin{array}{cc} \; & \;\;\;\;\;\;\;\;\;\;\;\;\;\;\;\;\;\;\;\;\;\;\;\;\;\;\;\;\;\leq I\left(X;Z^{n}\right)-H\left(\tilde{M}|C_{n}\right)+H\left(\tilde{M}|Z^{n}MC_{n}\right) \end{array}$$

(51e) $$\begin{array}{cc} \; & \;\;\;\;\;\;\;\;\;\;\;\;\;\;\;\;\;\;\;\;\;\;\;\;\;\;\;\;\;\;\;\;\;\;\;\;\;\;\;=h\left(Z^{n}\right)-h\left(N_{Z}^{n}\right)-H\left(\tilde{M}|C_{n}\right)+H\left(\tilde{M}|Z^{n}MC_{n}\right) \end{array}$$

(51f) $$\begin{array}{cc} \; & \;\;\;\;\;\;\;\;\;\;\;\;\;\;\;\;\;\;\;\;\;\;\;\;\;\;\;\;\;\;\;\;\;\;\;\;\;\;\;\;\leq \frac{1}{2}\log\left|GK_{X}G^{T}+I_{n}\right|-H\left(\tilde{M}|C_{n}\right)+H\left(\tilde{M}|Z^{n}MC_{n}\right) \end{array}$$

(51g) $$\begin{array}{cc} \; & \;\;\;\;\;\;\;\;\;\;\;\;\;\;\;\;\;\;\;\;\;\;\;\;\;\;\;\;\;\;\;\;\;\;\;\;\;\;\;\;\;\;=\frac{n}{2}\log\left(1+h_{1}P_{1}+h_{2}P_{2}\right)-H\left(\tilde{M}|C_{n}\right)+H\left(\tilde{M}|Z^{n}MC_{n}\right) \end{array}$$

(51h) $$\begin{array}{cc} \; & \;\;\;\;\;\;\;\;\;\;\;\;\;\;\;\;\;\;\;\;\;\;\;\;\;\;\;\;\;\;\;=nI\left(X_{1}X_{2};Z\right)-H\left(\tilde{M}|C_{n}\right)+H\left(\tilde{M}|Z^{n}MC_{n}\right) \end{array}$$

(51i) $$\begin{array}{cc} \; & \;\;\;\;\;\;\;\;\;\;\;\;\;\;\;\;\;\;\;\;\;\;\;\;\;\;\;\;\;\;\;\;\;\;\;\;\;\;\;\;\;\;\;\leq nI\left(X_{1}X_{2};Z\right)-n\left(I\left(X_{1}X_{2};Z\right)-2\delta \right)+O\left(nE_{C_{n}}\left[\tilde{e}\left(C_{n}\right)\right]\right) \end{array}$$

(51j) $$\begin{array}{cc} \; & \;\;\;\;\;\;\;\;\;\;\;=2\delta n+o(n), \end{array}$$

where Equation (51b) holds by independence between *M* and $\tilde{M}$; Equation (51c) holds because $\left(M,\tilde{M}\right)-\left(X,C_{n}\right)-Z^{n}$ forms a Markov chain; Equation (51d) holds because $C_{n}-X-Z^{n}$ forms a Markov chain; Equation (51f) holds because $h\left(N_{Z}^{n}\right)=\frac{1}{2}\log\left({\left(2\pi e\right)}^{n}\right)$ and because $h\left(Z^{n}\right)\leq \frac{1}{2}\log({\left(2\pi e\right)}^{n}|GK_{X}G^{T}+I_{n}\left|\right)$ by Equation (49) and the maximal differential entropy lemma (e.g., [31] Eq. (2.6)); Equation (51g) holds by Equation (50); in Equation (51i), we used the definition of ${\tilde{R}}_{1}+{\tilde{R}}_{2}$ and the uniformity of $\tilde{M}$ to obtain the second term, and Fano’s inequality to obtain the third term; Equation (51j) holds by Equation (48c).

Note that the idea of considering a fictitious decoder at the eavesdropper to use Fano’s inequality in Equation (51i) is a standard technique that already appeared in [32].

### 7.2. Case 2

We only consider Case 2.a; Case 2.b is handled by exchanging the role of the transmitters. Let $\underline{R}≜\left(R_{1},R_{2}\right)\in D\left(P_{1},P_{2}\right)$. There exists α∈[0,1[ such that $\underline{R}=\left(1-\alpha \right){\underline{C}}_{1}+\alpha {\underline{\tilde{C}}}_{2}$. The corner point ${\underline{C}}_{1}$ is achievable by Case 1, however, recall that since the first component of ${\underline{\tilde{C}}}_{2}$ is negative, it thus cannot be achieved as in Case 1, and one cannot perform time-sharing between ${\underline{C}}_{1}$ and ${\underline{\tilde{C}}}_{2}$ to achieve $\underline{R}$. Instead, we achieve $\underline{R}$ as follows. We define $k,k^{\prime }\in N$ such that $k^{\prime }/k={\left(1-\alpha \right)}^{-1}-1+\epsilon$, ϵ>0, this is possible by density of Q in R. We realize a first transmission $T_{1}$ as in Case 1 of a pair of confidential messages of length $nk{\underline{C}}_{1}$. Part of these confidential messages is dedicated to exchange a secret key of length $nk^{\prime }\left(I\left(X_{1};Z|X_{2}\right)-I\left(X_{1};Y\right)\right)>0$ between Transmitter 1 and the receiver, which is possible because $\left(1-\alpha \right){\underline{C}}_{1}+\alpha {\underline{\tilde{C}}}_{2}=\underline{R}$ has positive components. We then realize a second transmission $T_{2}$ of a pair of confidential messages of length $nk^{\prime }\left(0,I\left(X_{2};Y|X_{1}\right)-I\left(X_{2};Z\right)\right)$ assisted with the secret key that is shared between Transmitter 1 and the receiver. Hence, the overall transmission rate of confidential messages is $\frac{k}{k+k^{\prime }}{\underline{C}}_{1}+\frac{k^{\prime }}{k+k^{\prime }}{\underline{\tilde{C}}}_{2}$, which is arbitrarily close to $\underline{R}$ by choosing a sufficiently small ϵ. We now explain how transmission $T_{2}$ is performed. We repeat $k^{\prime }$ times the following coding scheme.

**Codebook construction**: Perform the same codebook construction as in Case 1 for Transmitter 2. For Transmitter 1, construct a codebook with $\left\lceil 2^{n{\overset{˘}{R}}_{1}}\right\rceil \left\lceil 2^{n{\overset{˚}{R}}_{1}}\right\rceil$ codewords drawn independently and uniformly on the sphere of radius $\sqrt{nP_{1}}$ in $R^{n}$. The codewords are labeled $x_{1}^{n}\left({\overset{˘}{m}}_{1},{\overset{˚}{m}}_{1}\right)$, where ${\overset{˘}{m}}_{1}\in ⟦1,2^{n{\overset{˘}{R}}_{1}}⟧$, ${\overset{˚}{m}}_{1}\in ⟦1,2^{n{\overset{˚}{R}}_{1}}⟧$. We define the rates ${\overset{˘}{R}}_{1}≜I\left(X_{1};Y\right)-\delta$, ${\overset{˚}{R}}_{1}≜I\left(X_{1};Z|X_{2}\right)-I\left(X_{1};Y\right)-\delta$, and ${\tilde{R}}_{1}≜{\overset{˘}{R}}_{1}+{\overset{˚}{R}}_{1}=I\left(X_{1};Z|X_{2}\right)-2\delta$.

**Encoding at Transmitters**: Encoding for Transmitter 2 is as in Case 1. Given $\left({\overset{˘}{m}}_{1},{\overset{˚}{m}}_{1}\right)$, Transmitter 1 forms $x_{1}^{n}\left({\overset{˘}{m}}_{1},{\overset{˚}{m}}_{1}\right)$, where ${\overset{˚}{m}}_{1}$ is seen as a secret key known at the receiver and that has been shared through transmission $T_{1}$ described above. In the following, we define ${\tilde{m}}_{1}≜\left({\overset{˘}{m}}_{1},{\overset{˚}{m}}_{1}\right)$.

**Decoding and average probability of error**: As in Case 1, using minimum distance decoding, one can show that on average over the codebooks, the receiver can reconstruct $x_{1}^{n}\left({\overset{˘}{m}}_{1},{\overset{˚}{m}}_{1}\right)$ with a vanishing average probability of error because ${\overset{˚}{m}}_{1}$ is known at the receiver and because ${\overset{˘}{R}}_{1}<I\left(X_{1};Y\right)$. The receiver can then reconstruct $x_{2}^{n}$ as in Case 1.

**Equivocation**: The equivocation computation for transmission $T_{2}$ is as in Case 1 by remarking that it is possible on average over the codebooks to reconstruct with vanishing average probability of error first $x_{2}^{n}$ given $\left(z^{n},m_{2}\right)$ and then $x_{1}^{n}$ given $\left(z^{n},x_{2}^{n}\right)$ by using that ${\tilde{R}}_{1}<I\left(X_{1};Z|X_{2}\right)$.

Finally, to conclude that $\underline{R}$ is achievable, we need to show that the secrecy constraint is satisfied for the joint transmissions $T_{1}$ and $T_{2}$. We use the superscript $\left(T_{i}\right)$ to denote random variables associated with transmission $T_{i}$, i∈{1,2}. Define $M^{\left(T_{1}\right)}≜\left(M_{1}^{\left(T_{1}\right)}\setminus {\overset{˚}{M}}_{1}^{\left(T_{1}\right)},M_{2}^{\left(T_{1}\right)}\right)$, the confidential messages sent during transmission $T_{1}$ excluding ${\overset{˚}{M}}_{1}^{\left(T_{1}\right)}$, defined as all the confidential messages sent during transmission $T_{1}$ and used during transmission $T_{2}$. We define $M^{\left(T_{2}\right)}≜\left(\emptyset ,M_{2}^{\left(T_{2}\right)}\right)$ as the confidential messages sent during transmission $T_{2}$. We define ${\tilde{M}}^{\left(T_{i}\right)}≜\left({\tilde{M}}_{1}^{\left(T_{i}\right)},{\tilde{M}}_{2}^{\left(T_{i}\right)}\right)$ as the randomization sequences used by both transmitters in Transmission $T_{i}$, i∈{1,2}. We also define $X^{\left(T_{i}\right)}$ as all the channel inputs from both transmitters in Transmission $T_{i}$, i∈{1,2}, and $Z^{\left(T_{i}\right)}$ as all the channel outputs observed by the eavesdropper in Transmission i∈{1,2}. Finally, we define $M^{\left(T_{1},T_{2}\right)}≜\left(M^{\left(T_{1}\right)},M^{\left(T_{2}\right)}\right)$, ${\tilde{M}}^{\left(T_{1},T_{2}\right)}≜\left({\tilde{M}}^{\left(T_{1}\right)},{\tilde{M}}^{\left(T_{2}\right)}\right)$, $Z^{\left(T_{1},T_{2}\right)}≜\left(Z^{\left(T_{1}\right)},Z^{\left(T_{2}\right)}\right)$, $X^{\left(T_{1},T_{2}\right)}≜\left(X^{\left(T_{1}\right)},X^{\left(T_{2}\right)}\right)$, $C_{n}^{\left(T_{1},T_{2}\right)}≜\left(C_{n}^{\left(T_{1}\right)},C_{n}^{\left(T_{2}\right)}\right)$. We have

$$\begin{array}{cc} \; & I(M^{\left(T_{1},T_{2}\right)};Z^{\left(T_{1},T_{2}\right)}|C_{n}^{\left(T_{1},T_{2}\right)}) \end{array}$$

(52a) $$\begin{array}{cc} \; & =I\left(M^{\left(T_{1},T_{2}\right)}{\tilde{M}}^{\left(T_{1},T_{2}\right)};Z^{\left(T_{1},T_{2}\right)}|C_{n}^{\left(T_{1},T_{2}\right)}\right)-I\left({\tilde{M}}^{\left(T_{1},T_{2}\right)};Z^{\left(T_{1},T_{2}\right)}|M^{\left(T_{1},T_{2}\right)}C_{n}^{\left(T_{1},T_{2}\right)}\right)\\ \; & =I\left(M^{\left(T_{1},T_{2}\right)}{\tilde{M}}^{\left(T_{1},T_{2}\right)};Z^{\left(T_{1},T_{2}\right)}|C_{n}^{\left(T_{1},T_{2}\right)}\right)-H\left({\tilde{M}}^{\left(T_{1},T_{2}\right)}|C_{n}^{\left(T_{1},T_{2}\right)}\right) \end{array}$$

(52b) −−+H(M˜(T1,T2)|Z(T1,T2)M(T1,T2)Cn(T1,T2))≤I(X(T1,T2);Z(T1,T2)|Cn(T1,T2))−H(M˜(T1,T2)|Cn(T1,T2))

(52c) −−+H(M˜(T1,T2)|Z(T1,T2)M(T1,T2)Cn(T1,T2))

(52d) $$\begin{array}{cc} \; & \leq I\left(X^{\left(T_{1},T_{2}\right)};Z^{\left(T_{1},T_{2}\right)}\right)-H\left({\tilde{M}}^{\left(T_{1},T_{2}\right)}|C_{n}^{\left(T_{1},T_{2}\right)}\right)+H\left({\tilde{M}}^{\left(T_{1},T_{2}\right)}|Z^{\left(T_{1},T_{2}\right)}M^{\left(T_{1},T_{2}\right)}C_{n}^{\left(T_{1},T_{2}\right)}\right) \end{array}$$

(52e) $$\begin{array}{cc} \; & \leq n\left(k+k^{\prime }\right)I\left(X_{1}X_{2};Z\right)-H\left({\tilde{M}}^{\left(T_{1},T_{2}\right)}|C_{n}^{\left(T_{1},T_{2}\right)}\right)+H\left({\tilde{M}}^{\left(T_{1},T_{2}\right)}|Z^{\left(T_{1},T_{2}\right)}M^{\left(T_{1},T_{2}\right)}C_{n}^{\left(T_{1},T_{2}\right)}\right) \end{array}$$

(52f) $$\begin{array}{cc} \; & \leq 3n\delta \left(k+k^{\prime }\right)+H\left({\tilde{M}}^{\left(T_{1},T_{2}\right)}|Z^{\left(T_{1},T_{2}\right)}M^{\left(T_{1},T_{2}\right)}C_{n}^{\left(T_{1},T_{2}\right)}\right) \end{array}$$

(52g) $$\begin{array}{cc} \; & \leq 3n\delta \left(k+k^{\prime }\right)+O\left(nE_{C_{n}^{\left(T_{1},T_{2}\right)}}\left[\tilde{e}\left(C_{n}^{\left(T_{1},T_{2}\right)}\right)\right]\right), \end{array}$$

where Equation (52b) holds because we defined $M^{\left(T_{1},T_{2}\right)}$ such that $M^{\left(T_{1},T_{2}\right)}$ is independent from ${\tilde{M}}^{\left(T_{1},T_{2}\right)}$, Equation (52c) holds because $\left(M^{\left(T_{1},T_{2}\right)},{\tilde{M}}^{\left(T_{1},T_{2}\right)}\right)-\left(C_{n}^{\left(T_{1},T_{2}\right)},X^{\left(T_{1},T_{2}\right)}\right)-Z^{\left(T_{1},T_{2}\right)}$ forms a Markov chain, Equation (52d) holds because $C_{n}^{\left(T_{1},T_{2}\right)}-X^{\left(T_{1},T_{2}\right)}-Z^{\left(T_{1},T_{2}\right)}$ forms a Markov chain, Equation (52e) holds similar to Equation (51h), Equation (52f) holds because by definition ${\tilde{R}}_{1}+{\tilde{R}}_{2}\geq I\left(X_{1}X_{2};Z\right)-3\delta$, Equation (52g) holds by Fano’s inequality with $\tilde{e}\left(C_{n}^{\left(T_{1},T_{2}\right)}\right)$ defined as the probability of error to reconstruct ${\tilde{M}}^{\left(T_{1},T_{2}\right)}$ given $\left(Z^{\left(T_{1},T_{2}\right)},M^{\left(T_{1},T_{2}\right)}\right)$ using minimum distance decoding as in Case 1. Then, define ${\tilde{e}}^{\left(1\right)}\left(C_{n}^{\left(T_{1},T_{2}\right)}\right)$ as the error probability to reconstruct ${\tilde{M}}^{\left(T_{2}\right)}$ from $\left(Z^{\left(T_{2}\right)},M^{\left(T_{2}\right)}\right)$ using minimum distance decoding, and ${\tilde{e}}^{\left(2\right)}\left(C_{n}^{\left(T_{1},T_{2}\right)}\right)$ as the error probability to reconstruct ${\tilde{M}}^{\left(T_{1}\right)}$ from $\left(Z^{\left(T_{1}\right)},M^{\left(T_{1}\right)},{\tilde{M}}^{\left(T_{2}\right)}\right)$ using minimum distance decoding. As in the analysis of Case 1 and by observing that ${\overset{˚}{M}}_{1}^{\left(T_{1}\right)}$ is included in ${\tilde{M}}^{\left(T_{2}\right)}$, we have

(53a) $$\begin{array}{cc} E_{C_{n}^{\left(T_{1},T_{2}\right)}}\left[\tilde{e}\left(C_{n}^{\left(T_{1},T_{2}\right)}\right)\right]\; & \leq E_{C_{n}^{\left(T_{1},T_{2}\right)}}\left[{\tilde{e}}^{\left(1\right)}\left(C_{n}^{\left(T_{1},T_{2}\right)}\right)\right]+E_{C_{n}^{\left(T_{1},T_{2}\right)}}\left[{\tilde{e}}^{\left(2\right)}\left(C_{n}^{\left(T_{1},T_{2}\right)}\right)\right] \end{array}$$

(53b) $$\begin{array}{cc} \; & \overset{n\to \infty }{\to }0. \end{array}$$

We conclude from Equations (52g) and (53b)

(54) $$\begin{array}{c} I\left(M^{\left(T_{1},T_{2}\right)};Z^{\left(T_{1},T_{2}\right)}|C_{n}^{\left(T_{1},T_{2}\right)}\right)=3n\delta \left(k+k^{\prime }\right)+o\left(n\right). \end{array}$$

### 7.3. Case 3

We have $I\left(X_{1};Z|X_{2}\right)-I\left(X_{1};Y\right)>0$ and $I\left(X_{2};Z|X_{1}\right)-I\left(X_{2};Y\right)>0$ as depicted in Figure 5. Assume $I\left(X_{1}X_{2};Y\right)-I\left(X_{1}X_{2};Z\right)>0$, otherwise $R_{1,2}^{MAC}\left(P_{1},P_{2}\right)=\left\{\left(0,0\right)\right\}$. We will use the following lemma.

**Lemma** **2.** *Define $h_{\Lambda }≜{\left(1+\Lambda \right)}^{-1}$. We have*

1.
$I\left(X_{1};Z|X_{2}\right)-I\left(X_{1};Y\right)\leq I\left(X_{1};Y|X_{2}\right)-I\left(X_{1};Z\right)$ *or $I\left(X_{2};Z|X_{1}\right)-I\left(X_{2};Y\right)\leq I\left(X_{2};Y|X_{1}\right)-I\left(X_{2};Z\right)$.*
2. *$h_{1}<h_{\Lambda }$ or $h_{2}<h_{\Lambda }$.*
3. Assume $I\left(X_{1};Z|X_{2}\right)-I\left(X_{1};Y\right)\leq I\left(X_{1};Y|X_{2}\right)-I\left(X_{1};Z\right)$. There exists $m,m^{\prime }\in N^{*}$, such that
(55a) $$\begin{array}{cc} m^{\prime }\left(I\left(X_{1};Y|X_{2}\right)-I\left(X_{1};Z\right)\right) & \geq m(I\left(X_{1};Z|X_{2}\right)-I\left(X_{1};Y\right)), \end{array}$$
(55b) $$\begin{array}{cc} m(I\left(X_{2};Y|X_{1}\right)-I\left(X_{2};Z\right)) & >m^{\prime }\left(I\left(X_{2};Z|X_{1}\right)-I\left(X_{2};Y\right)\right). \end{array}$$

**Proof.**  (i) Assume that

(56a) $$\begin{array}{cc} I\left(X_{1};Z|X_{2}\right)-I\left(X_{1};Y\right) & >I\left(X_{1};Y|X_{2}\right)-I\left(X_{1};Z\right), \end{array}$$

(56b) $$\begin{array}{cc} I\left(X_{2};Z|X_{1}\right)-I\left(X_{2};Y\right) & >I\left(X_{2};Y|X_{1}\right)-I\left(X_{2};Z\right). \end{array}$$

Then,

(57) $$\begin{array}{cc} \; & I\left(X_{1};Z|X_{2}\right)-I\left(X_{1};Y\right)+I\left(X_{2};Z|X_{1}\right)-I\left(X_{2};Y\right)\\ \; & >I\left(X_{1};Y|X_{2}\right)-I\left(X_{1};Z\right)+I\left(X_{2};Y|X_{1}\right)-I\left(X_{2};Z\right), \end{array}$$

which contradicts the fact that $I\left(X_{1};Z|X_{2}\right)-I\left(X_{1};Y\right)<I\left(X_{2};Y|X_{1}\right)-I\left(X_{2};Z\right)$ and $I\left(X_{2};Z|X_{1}\right)-I\left(X_{2};Y\right)<I\left(X_{1};Y|X_{2}\right)-I\left(X_{1};Z\right)$.

(ii) By contradiction, if $h_{1}\geq h_{\Lambda }$ and $h_{2}\geq h_{\Lambda }$, then $I\left(X_{1}X_{2};Y\right)-I\left(X_{1}X_{2};Z\right)\leq 0$.

(iii) Choose $m^{\prime }\in N^{*}$ such that

(58) $$\begin{array}{c} I\left(X_{1};Z|X_{2}\right)-I\left(X_{1};Y\right)\leq m^{\prime }\left(I\left(X_{1}X_{2};Y\right)-I\left(X_{1}X_{2};Z\right)\right). \end{array}$$

Then, there exists $m\in N^{*}$ and $r\in [0,I\left(X_{1};Z|X_{2}\right)-I\left(X_{1};Y\right)[$ such that

(59) $$\begin{array}{c} m^{\prime }\left(I\left(X_{1};Y|X_{2}\right)-I\left(X_{1};Z\right)\right)=m\left(I\left(X_{1};Z|X_{2}\right)-I\left(X_{1};Y\right)\right)+r. \end{array}$$

Then, we have

$$\begin{array}{cc} \; & m(I\left(X_{2};Y|X_{1}\right)-I\left(X_{2};Z\right)) \end{array}$$

(60a) $$\begin{array}{cc} \; & =m\left(I\left(X_{1};Z|X_{2}\right)-I\left(X_{1};Y\right)\right)+m\left(I\left(X_{1}X_{2};Y\right)-I\left(X_{1}X_{2};Z\right)\right) \end{array}$$

(60b) $$\begin{array}{cc} \; & =m^{\prime }\left(I\left(X_{1};Y|X_{2}\right)-I\left(X_{1};Z\right)\right)+m\left(I\left(X_{1}X_{2};Y\right)-I\left(X_{1}X_{2};Z\right)\right)-r \end{array}$$

(60c) $$\begin{array}{cc} \; & =m^{\prime }\left(I\left(X_{2};Z|X_{1}\right)-I\left(X_{2};Y\right)\right)+\left(m+m^{\prime }\right)\left(I\left(X_{1}X_{2};Y\right)-I\left(X_{1}X_{2};Z\right)\right)-r \end{array}$$

(60d) $$\begin{array}{cc} \; & >m^{\prime }\left(I\left(X_{2};Z|X_{1}\right)-I\left(X_{2};Y\right)\right)+m\left(I\left(X_{1}X_{2};Y\right)-I\left(X_{1}X_{2};Z\right)\right) \end{array}$$

(60e) $$\begin{array}{cc} \; & >m^{\prime }\left(I\left(X_{2};Z|X_{1}\right)-I\left(X_{2};Y\right)\right), \end{array}$$

where Equation (60b) holds by Equation (59), and Equation (60d) holds because $r<I\left(X_{1};Z|X_{2}\right)-I\left(X_{1};Y\right)\leq m^{\prime }\left(I\left(X_{1}X_{2};Y\right)-I\left(X_{1}X_{2};Z\right)\right)$. □

By (i) in Lemma 2, assume without loss of generality that $I\left(X_{1};Z|X_{2}\right)-I\left(X_{1};Y\right)\leq I\left(X_{1};Y|X_{2}\right)-I\left(X_{1};Z\right)$ by exchanging the role of the transmitters if necessary. We let *m*, $m^{\prime }$ be as in (iii) of Lemma 2. $D(P_{1},P_{2})$ is achieved in four steps.

**Step 1**. During a first transmission $T_{0}$, Transmitter 2 transmits a confidential message of length $nm^{\prime }\left(I\left(X_{2};Z|X_{1}\right)-I\left(X_{2};Y\right)\right)$ to the receiver. This is possible with a point-to-point wiretap code; as in Case 1, when Transmitter 1 remains silent and when $h_{\Lambda }>h_{2}$. If, on the other hand, $h_{\Lambda }\leq h_{2}$, then by (ii) in Lemma 2, $h_{\Lambda }>h_{1}$ and Transmitter 2 can transmit a confidential message of length $nm^{\prime }\left(I\left(X_{2};Z|X_{1}\right)-I\left(X_{2};Y\right)\right)$ as follows. Transmitter 1 transmits a confidential message of length $nk(I\left(X_{1};Z|X_{2}\right)-I\left(X_{1};Y\right))$, where $k\in N^{*}$ is such that $nk\left(I\left(X_{2};Y|X_{1}\right)-I\left(X_{2};Z\right)\right)\geq nm^{\prime }\left(I\left(X_{2};Z|X_{1}\right)-I\left(X_{2};Y\right)\right)$. Using this secret key shared by Transmitter 1 and the receiver, Transmitter 2 can transmit a confidential message of length $nk(I\left(X_{2};Y|X_{1}\right)-I\left(X_{2};Z\right))$ as in Case 2. Note that Step 1 is operated in a fixed number of blocks of length *n*.

**Step 2**. As in Case 2, the transmitters achieve transmission $T_{1}$ of confidential messages of length $\left(nm^{\prime }\left(I\left(X_{1};Y|X_{2}\right)-I\left(X_{1};Z\right)\right),0\right)$ by using the secret key exchanged during $T_{0}$ between Transmitter 2 and the receiver. Then, as in Case 2 and because $m^{\prime }\left(I\left(X_{1};Y|X_{2}\right)-I\left(X_{1};Z\right)\right)-m\left(I\left(X_{1};Z|X_{2}\right)-I\left(X_{1};Y\right)\right)\geq 0$ by (iii) in Lemma 2, the transmitters achieve a transmission $T_{2}$ of confidential messages of length $\left(0,nm\left(I\left(X_{2};Y|X_{1}\right)-I\left(X_{2};Z\right)\right)\right)$ using a secret key of length $nm(I\left(X_{1};Z|X_{2}\right)-I\left(X_{1};Y\right))$ exchanged between Transmitter 1 and the receiver during $T_{1}$. Hence, after $T_{1}$ and $T_{2}$, the transmitters achieved the transmission of confidential messages of length $\left(nm^{\prime }\left(I\left(X_{1};Y|X_{2}\right)-I\left(X_{1};Z\right)\right)-nm\left(I\left(X_{1};Z|X_{2}\right)-I\left(X_{1};Y\right)\right),nm\left(I\left(X_{2};Y|X_{1}\right)-I\left(X_{2};Z\right)\right)\right)$.

**Step 3**. The transmitters repeat $T_{1}$ and $T_{2}$ *t* times, where *t* is arbitrary, since $m\left(I\left(X_{2};Y|X_{1}\right)-I\left(X_{2};Z\right)\right)-m^{\prime }\left(I\left(X_{2};Z|X_{1}\right)-I\left(X_{2};Y\right)\right)>0$ by (iii) in Lemma 2. After these *t* repetitions, the rate pair achieved is arbitrarily close to

(61) $$\begin{array}{c} \underline{R}=\frac{1}{m+m^{\prime }}(m^{\prime }\left(I\left(X_{1};Y|X_{2}\right)-I\left(X_{1};Z\right)\right)-m\left(I\left(X_{1};Z|X_{2}\right)-I\left(X_{1};Y\right)\right),\\ m\left(I\left(X_{2};Y|X_{1}\right)-I\left(X_{2};Z\right)\right)-m^{\prime }\left(I\left(X_{2};Z|X_{1}\right)-I\left(X_{2};Y\right)\right)) \end{array}$$

provided that *t* is large enough since Step 1 only requires a fixed number of transmission blocks. Observe that $\underline{R}\in D\left(P_{1},P_{2}\right)$.

**Step 4**. Any point of $D(P_{1},P_{2})$ can then be achieved as in Case 2 by doing the substitutions ${\underline{C}}_{1}\leftarrow \underline{R}$ and ${\underline{C}}_{2}\leftarrow \underline{R}$ in Case 2.a and Case 2.b, respectively.

The proof that secrecy holds over the joint transmissions is similar to Case 2 and thus omitted.

## 8. Proof of Theorem 3

We first show that determining a converse for our model reduces to determining a converse for a similar model when the jammer is inactive, i.e., when Λ=0.

**Lemma** **3.** *Let $O≜\left\{\left(R_{1},R_{2}\right):R_{1}\leq B_{1},R_{2}\leq B_{2},R_{1}+R_{2}\leq B_{1,2}\right\}$ be an outer bound, i.e., a set that contains all possibly achievable rate pairs, for the Gaussian MAC-WT-JA with parameters $\left(\Gamma_{1},\Gamma_{2},h_{1},h_{2},0,\sigma_{Y}^{2}+\Lambda ,\sigma_{Z}^{2}\right)$. Then,*

$$\left\{\left(R_{1},R_{2}\right):R_{1}\leq \left\{\begin{array}{cc} B_{1} & \;\mathrm{if}\;\Gamma_{1}>\Lambda \\ 0 & \;\mathrm{if}\;\Gamma_{1}\leq \Lambda \end{array}\right.,R_{2}\leq \left\{\begin{array}{cc} B_{2} & \;\mathrm{if}\;\Gamma_{2}>\Lambda \\ 0 & \;\mathrm{if}\;\Gamma_{2}\leq \Lambda \end{array}\right.,R_{1}+R_{2}\leq B_{1,2}\right\}$$

*is an outer bound for the Gaussian MAC-WT-JA with parameters $\left(\Gamma_{1},\Gamma_{2},h_{1},h_{2},\Lambda ,\sigma_{Y}^{2},\sigma_{Z}^{2}\right)$.*

**Proof.**  Consider any encoders and decoder for the Gaussian MAC-WT-JA with the parameters $\left(\Gamma_{1},\Gamma_{2},h_{1},h_{2},\Lambda ,\sigma_{Y}^{2},\sigma_{Z}^{2}\right)$ that achieve the rate pair $\left(R_{1},R_{2}\right)$. Note that by [24] [Theorem 2.3], for any l∈{1,2} such that $\Gamma_{l}\leq \Lambda$, we must have $R_{l}=0$, since an outer bound for the model in [24] is also an outer bound for the Gaussian MAC-WT-JA, which has the additional security constraint (2b). Then, to derive an outer bound, it is sufficient to consider a specific jamming strategy and study the best achievable rates for this jamming strategy, since the boundaries of the capacity region correspond to the best (from the jammer’s point of view) jamming strategies and any other jamming strategy can only enlarge the set of achievable rates. We assume that in each transmission block, the jamming sequence is $S^{n}$ with the components independent and identically distributed according to a zero-mean Gaussian random variable with the variance $\Lambda^{\prime }<\Lambda$. The average probability of error at the legitimate receiver is thus upper-bounded by $\sup_{S\in S}P\left[\hat{M}\neq M\right]+kP\left[{∥S^{n}∥}^{2}>n\Lambda \right]\overset{n\to \infty }{\to }0$ where we used the notation of Definition 1 and the fact that $kP\left[{∥S^{n}∥}^{2}>n\Lambda \right]\overset{n\to \infty }{\to }0$ since $\Lambda^{\prime }<\Lambda$. Hence, since the secrecy constraint is independent of $\Lambda^{\prime }$, we obtain the reliability and secrecy constraints for a Gaussian MAC-WT-JA with parameters $\left(\Gamma_{1},\Gamma_{2},h_{1},h_{2},0,\sigma_{Y}^{2}+\Lambda^{\prime },\sigma_{Z}^{2}\right)$, meaning that $\left(R_{1},R_{2}\right)\in O^{\prime }$, where $O^{\prime }$ is an outer bound for the Gaussian MAC-WT-JA with parameters $\left(\Gamma_{1},\Gamma_{2},h_{1},h_{2},0,\sigma_{Y}^{2}+\Lambda^{\prime },\sigma_{Z}^{2}\right)$. Finally, we conclude the proof by choosing $\Lambda^{\prime }$ arbitrarily close to Λ. □

We now obtain Theorem 3 as follows. (i) holds from Lemma 3. (ii) holds from Lemma 3 and [33] [Theorem 6] by remarking that $x\mapsto \log\left(\frac{1+x{\left(1+\Lambda \right)}^{-1}}{1+xh}\right)$ is non-decreasing when ${\left(1+\Lambda \right)}^{-1}>h$ and negative when ${\left(1+\Lambda \right)}^{-1}\leq h$.

## 9. Concluding Remarks

In this paper, we defined Gaussian wiretap channels in the presence of an eavesdropper aided by a jammer. The jamming signal is power-constrained and assumed to be oblivious of the legitimate users’ communication but is not restricted to be Gaussian. We studied several models in this framework, namely point-to-point, multiple-access, broadcast, and symmetric interference settings. We derived inner and outer bounds for these settings, and identified conditions for these bounds to coincide. We stress that no shared randomness among the legitimate users is required in our coding schemes.

Our achievability scheme for the Gaussian MAC-WT-JA relies on novel time-sharing strategies and an extension of successive decoding for multiple-access channels to multiple-access wiretap channels via secret-key exchanges. An open problem remains to provide a scheme that avoids time-sharing. Section 4.2 provides such a scheme for some rate pairs and channel parameters; however, it might not be possible to achieve the entire region of Theorem 2 by solely relying on point-to-point codes, in which case the design of multi-transmitter codes for arbitrarily varying multiple-access channels would be necessary.

Finally, beyond proving the existence of achievability schemes for our models, finding explicit coding schemes largely remains an open problem. We note that [34] investigates this problem for short communication blocklengths over point-to-point channels via a practical approach that relies on deep learning. Another open problem is to achieve the same regions as that derived in this paper under strong and semantic security guarantees.

## Data Availability

Not applicable.

## Appendix A. Supporting Results

**Lemma** **A1** ([1]). *Let ϵ>0, $\eta \in ]8\sqrt{\epsilon },1[$, K>2ϵ, R∈[2ϵ,K], and $N≜e^{nR}$. Let $X_{1}^{n},\ldots ,X_{N}^{n}$ be independent random variables uniformly distributed on the unit sphere. With probability arbitrarily close to one as n→∞, we have*

1. *$|\left\{j:\langle X_{j}^{n},u^{n}\rangle \geq \alpha \right\}|\leq e^{n\left({\left[R+\frac{1}{2}\log\left(1-\alpha^{2}\right)\right]}^{+}+\epsilon \right)}$ for any unit vector $u^{n}\in R^{n}$, α>0.*
2. *$\frac{1}{N}\left|\{i:\right|\langle X_{j}^{n},X_{i}^{n}\rangle \left|\geq \alpha ,\right|\langle X_{j}^{n},u^{n}\rangle |\geq \beta ,\;\mathrm{for}\;\mathrm{some}\;j\neq i\}|\leq e^{-n\epsilon }$ for any unit vector $u^{n}\in R^{n}$, α,β∈[0,1] such that α≥η, $\alpha^{2}+\beta^{2}>1+\eta -e^{-2R}$.*

**Theorem** **A1** ([1,24]). *Consider a channel whose output is defined as $Y^{n}=X^{n}+V^{n}+s^{n}$, where $X^{n}$ is the input such that ${∥X^{n}∥}^{2}\leq n$, $V^{n}$ represents noise (to be defined next), and $s^{n}$ is a state unknown to the encoder and decoder such that ${∥s^{n}∥}^{2}\leq n\Lambda$, Λ<1. Let σ,δ>0. Consider a codebook $C_{n}$ made of $N≜e^{n(\frac{1}{2}\log\left(1+{\left(\Lambda +\sigma^{2}\right)}^{-1}\right)-\delta )}$ codewords $\left(x_{1}^{n},\ldots ,x_{N}^{n}\right)$ that satisfy the two conditions of Lemma A1, and define the average probability of error $e(C_{n})$ of a minimum distance decoder as $e\left(C_{n}\right)≜\frac{1}{N}\sum_{i=1}^{N}P\left[{∥x_{i}^{n}+s^{n}+V^{n}-x_{j}^{n}∥}^{2}\leq {∥s^{n}+V^{n}∥}^{2},\;\mathrm{for}\;\mathrm{some}\;j\neq i\right]$.*

1. *(From [1]). If $V^{n}$ is a vector with i.i.d. zero-mean Gaussian coordinates with variance $\sigma^{2}$, then $\lim_{n\to \infty }e\left(C_{n}\right)=0$.*
2. *(From [24]). If $V^{n}≜W^{n}+U$, where $W^{n}$ is a vector with i.i.d. zero-mean Gaussian coordinates with variance $a^{2}$ and U is independently distributed uniformly at random on a sphere with radius $\sqrt{nb^{2}}$ such that $a^{2}+b^{2}=\sigma^{2}$, then $\lim_{n\to \infty }e\left(C_{n}\right)=0$.*

## Appendix B. Proof of Theorem 4

We first recall some definitions and results on polymatroids.

**Definition** **A1** ([35]). *Let $f:2^{M}\to R$. $P\left(f\right)≜\left\{{\left(R_{i}\right)}_{i\in M}\in R^{M}:R_{S}\leq f\left(S\right),\forall S\subseteq M\right\}$ associated with the function f is an* extended polymatroid *if f is submodular, i.e., ∀S,T⊆M,f(S∪T)+f(S∩T)≤f(S)+f(T).*

**Property** **A1** ([29] [Property 1]). *Define $g:2^{L(\Lambda )}\to R,T\mapsto I\left(X_{T};Y|X_{T^{c}}\right)-I\left(X_{T};Z\right)$, where $Y≜\sum_{l\in L(\Lambda )}X_{l}+N_{Y}$, $Z≜\sum_{l\in L(\Lambda )}\sqrt{h_{l}}X_{l}+N_{Z}$, with ${\left(X_{l}\right)}_{l\in L(\Lambda )}$, $N_{Y}$, $N_{Z}$ independent zero-mean Gaussian random variables with variances ${\left(P_{l}\right)}_{l\in L(\Lambda )}$, (1+Λ), 1, respectively.*

(A1) $$\begin{array}{c} C\left(\Lambda \right)≜\left\{{\left(R_{l}\right)}_{l\in L(\Lambda )}\in R^{\left|L(\Lambda )\right|}:\forall T\subseteq L\left(\Lambda \right),R_{T}\leq g\left(T\right)\right\} \end{array}$$

*associated with g is an extended polymatroid.*

**Property** **A2** ([35]). *Define the dominant face D(Λ) of C(Λ) as*

(A2) $$\begin{array}{c} D\left(\Lambda \right)≜\left\{{\left(R_{l}\right)}_{l\in L(\Lambda )}\in C\left(\Lambda \right):R_{L(\Lambda )}=g\left(L\left(\Lambda \right)\right)\right\}. \end{array}$$

*For π∈Sym(|L(Λ)|), where Sym(|L(Λ)|) is the symmetric group on L(Λ), for i,j∈L(Λ), define $\pi^{i:j}≜{\left(\pi (k)\right)}_{k\in ⟦i,j⟧}.$ D(Λ) is the convex hull of the vertices $V≜\left\{{\left(C_{\pi (i)}\right)}_{i\in ⟦1,|L(\Lambda )|⟧}:\pi \in \mathrm{Sym}(|L\left(\Lambda \right)\left|\right)\right\}$, where for π∈Sym(|L(Λ)|), for i∈⟦1,|L(Λ)|⟧, $C_{\pi (i)}=g\left(\{\pi^{i:|L(\Lambda )|}\}\right)-g\left(\{\pi^{i+1:|L(\Lambda )|}\}\right).$*

Define $D_{+}\left(\Lambda \right)≜D\left(\Lambda \right)\cap R_{+}^{\left|L(\Lambda )\right|}$. By Property A2, for any $\underline{R}\in D_{+}\left(\Lambda \right)$, for any $\underline{V}={\left(V_{l}\right)}_{l\in L(\Lambda )}\in V$, there exists $\alpha_{\underline{V}}\in \left[0,1\right]$, such that $\sum_{\underline{V}\in V}\alpha_{\underline{V}}=1$ and $\underline{R}=\sum_{\underline{V}\in V}\alpha_{\underline{V}}\underline{V}$. As remarked in [29], *g* is, in general, not non-decreasing; hence, some $\underline{V}\in V$ might have negative components and the successive decoding method [5] [Appendix C] cannot be applied to the multiple-access wiretap channel. We show in the following how to overcome this issue. For l∈L(Λ), define $R_{l}^{*}≜-\sum_{\underline{V}\in V}\alpha_{\underline{V}}𝟙\left\{V_{l}<0\right\}V_{l}$, and ${\underline{R}}^{*}≜{\left(R_{l}^{*}\right)}_{l\in L(\Lambda )}$. Our coding scheme operates in three steps, the idea of which is described below.

**Step 1.** For l∈L(Λ), a secret message of length $nR_{l}^{*}$ is exchanged between Transmitter *l* and the receiver.

**Step 2.** For all $\underline{V}\in V$, secret messages of length $n{\left(\alpha_{\underline{V}}𝟙\left\{V_{l}>0\right\}V_{l}\right)}_{l\in L(\Lambda )}$ are exchanged between the transmitters and the receiver, provided that secret sequences of length $n{\underline{R}}^{*}$ are shared between the transmitters and the receiver, which is ensured by Step 1. The overall length of secret communication is $n{\left(\sum_{\underline{V}\in V}\alpha_{\underline{V}}𝟙\left\{V_{l}>0\right\}V_{l}\right)}_{l\in L(\Lambda )}$, i.e., $n(\underline{R}+{\underline{R}}^{*})$.

**Step 3.** Repeat *t* times Step 2. It is possible to do so because secret sequences of length at least $n{\underline{R}}^{*}$ were exchanged between the transmitters and the receiver in Step 2. The overall rate of secret sequences exchanged between the transmitters and the receiver is thus $\underline{R}$, provided that *t* is large enough, since Step 1 only requires the transmission of a finite number of blocks.

The coding schemes and their analyses to realize Steps 1 and 2 are described in Appendix B.1 and Appendix B.2, respectively. In the remainder of the section, *Y* and *Z* are defined as in Property A1 with ${\left(X_{l}\right)}_{l\in L(\Lambda )}$ zero-mean Gaussian random variables with variances ${\left(P_{l}\right)}_{l\in L(\Lambda )}$.

### Appendix B.1. Proof of Step 1

The proof of Step 1 directly follows from the point-to-point setting, i.e., Theorem 1, applied to each l∈L(Λ) since we assumed $h_{l}<h_{\Lambda }$.

### Appendix B.2. Proof of Step 2

We fix $\underline{V}\in V$. The following procedure must be reiterated for each $\underline{V}\in V$ by applying a permutation π∈Sym(|L(Λ)|) on the labeling of the transmitters. For convenience, we relabel the transmitter from 1 to |L(Λ)| and redefine L(Λ) as ⟦1,|L(Λ)|⟧. We show how to exchange secret messages with rate ${\left(𝟙\left\{V_{l}>0\right\}V_{l}\right)}_{l\in L(\Lambda )}$ between the transmitters and the receiver, when they have access to pre-shared secrets (obtained from Step 1) with rate ${\left(-𝟙\left\{V_{l}<0\right\}V_{l}\right)}_{l\in L(\Lambda )}$. Define $I≜\{l\in L\left(\Lambda \right):V_{l}\leq 0\}$ and $I^{c}≜L\left(\Lambda \right)\setminus I$. We also use the notation $X_{L(\Lambda )}≜{\left(X_{l}\right)}_{l\in L(\Lambda )}$, $X_{L(\Lambda )}^{n}≜{\left(X_{l}^{n}\right)}_{l\in L(\Lambda )}$, and for i,j∈L(Λ), $X_{i:j}≜{\left(X_{l}\right)}_{l\in ⟦i,j⟧}$.

**Codebook construction**: For Transmitter $i\in I^{c}$, construct a codebook $C_{n}^{\left(i\right)}$ with $\left\lceil 2^{nR_{i}}\right\rceil \left\lceil 2^{n{\tilde{R}}_{i}}\right\rceil$ codewords drawn independently and uniformly on the sphere of radius $\sqrt{nP_{i}}$ in $R^{n}$. The codewords are labeled $x_{i}^{n}\left(m_{i},{\tilde{m}}_{i}\right)$, where $m_{i}\in ⟦1,2^{nR_{i}}⟧$, ${\tilde{m}}_{i}\in ⟦1,2^{n{\tilde{R}}_{i}}⟧$. We choose the rates as $R_{i}≜I\left(X_{i};Y|X_{1:i-1}\right)-I\left(X_{i};Z|X_{i+1:|L(\Lambda )|}\right)-\delta$, ${\tilde{R}}_{i}≜I\left(X_{i};Z|X_{i+1:|L(\Lambda )|}\right)-\delta$. For Transmitter i∈I, construct a codebook $C_{n}^{\left(i\right)}$ with $\left\lceil 2^{n{\overset{˘}{R}}_{i}}\right\rceil \left\lceil 2^{n{\overset{˚}{R}}_{i}}\right\rceil$ codewords drawn independently and uniformly on the sphere of radius $\sqrt{nP_{i}}$ in $R^{n}$. The codewords are labeled $x_{i}^{n}\left({\overset{˘}{m}}_{i},{\overset{˚}{m}}_{i}\right)$, where ${\overset{˘}{m}}_{i}\in ⟦1,2^{n{\overset{˘}{R}}_{i}}⟧$, ${\overset{˚}{m}}_{i}\in ⟦1,2^{n{\overset{˚}{R}}_{i}}⟧$. We define the rates ${\overset{˘}{R}}_{i}≜I\left(X_{i};Y|X_{1:i-1}\right)-\delta$, ${\overset{˚}{R}}_{i}≜I\left(X_{i};Z|X_{i+1:|L(\Lambda )|}\right)-I\left(X_{i};Y|X_{1:i-1}\right)-\delta$, and ${\tilde{R}}_{i}≜{\overset{˘}{R}}_{i}+{\overset{˚}{R}}_{i}=I\left(X_{i};Z|X_{i+1:|L(\Lambda )|}\right)-2\delta$. Define $C_{n}≜{\left(C_{n}^{\left(i\right)}\right)}_{i\in L(\Lambda )}$.

**Encoding at the transmitters**: For Transmitter $i\in I^{c}$, given $\left(m_{i},{\tilde{m}}_{i}\right)$, transmit $x_{i}^{n}\left(m_{i},{\tilde{m}}_{i}\right)$. For Transmitter i∈I, given $\left({\overset{˘}{m}}_{i},{\overset{˚}{m}}_{i}\right)$, transmit $x_{i}^{n}\left({\overset{˘}{m}}_{i},{\overset{˚}{m}}_{i}\right)$, where ${\overset{˚}{m}}_{i}$ is assumed to be known at the receiver by the transmissions in Step 1. In the following, we define for i∈I, ${\tilde{m}}_{i}≜\left({\overset{˘}{m}}_{i},{\overset{˚}{m}}_{i}\right)$. By convention, define for i∈I, $m_{i}≜\emptyset$. Also define $m≜{\left(m_{i}\right)}_{i\in L(\Lambda )}$, $\tilde{m}≜{\left({\tilde{m}}_{i}\right)}_{i\in L(\Lambda )}$. In the following, we refer to $\tilde{m}$ as randomization sequence.

**Decoding**: The receiver performs minimum distance decoding, i.e., given $y^{n}$, determine starting from i=1 to i=|L(Λ)|, $\left({\hat{m}}_{i},{\hat{\tilde{m}}}_{i}\right)≜\phi_{i}\left(y^{n},\sum_{j=1}^{i-1}x_{j}^{n}\left({\hat{m}}_{j},{\hat{\tilde{m}}}_{j}\right)\right)$ where

(A3) ϕi:(yn,x)↦(mi,m˜i)if∥yn−x−xin(mi,m˜i)∥2<∥yn−x−xin(mi′,m˜i′)∥2−−−−−−−−−−−−for(mi′,m˜i′)≠(mi,m˜i)0ifnosuch(mi,m˜i)∈⟦1,2nRi⟧×⟦1,2nR˜i⟧exists.

Define $\hat{m}≜{\left({\hat{m}}_{i}\right)}_{i\in L(\Lambda )}$, $\hat{\tilde{m}}≜{\left({\hat{\tilde{m}}}_{i}\right)}_{i\in L(\Lambda )}$. Let $e\left(C_{n},s^{n}\right)≜P\left[\hat{M}\neq M|C_{n}\right]$, we now prove that on average on $C_{n}$, we have $E_{C_{n}}\left[\sup_{s^{n}}e\left(C_{n},s^{n}\right)\right]+\frac{1}{n}I\left(M;Z^{n}|C_{n}\right)\overset{n\to \infty }{\to }0$. We will thus conclude that there exists a sequence of realizations $\left(C_{n}\right)$ of $\left(C_{n}\right)$ such that both $\sup_{s^{n}}e\left(C_{n},s^{n}\right)$ and $\frac{1}{n}I\left(M;Z^{n}|C_{n}\right)$ can be made arbitrarily close to zero as n→∞.

**Average probability of error**: We have

(A4a) $$\begin{array}{cc} e(C_{n},s^{n})\; & \leq P\left[\left.\hat{M}\neq M\;\mathrm{or}\;\hat{\tilde{M}}\neq \tilde{M}\right|C_{n}\right] \end{array}$$

(A4b) $$\begin{array}{cc} \; & \;\;\;\;\;\;\;\;\;\;\;\;\;\;\;\;\;\;\;\;\;\;\;\;\;\;\;\;=\sum_{i\in L(\Lambda )}e_{i}\left(C_{n},s^{n},\sum_{j=i+1}^{\left|L(\Lambda )\right|}x_{j}^{n}\left(M_{j},{\tilde{M}}_{j}\right)\right), \end{array}$$

where for i∈L(Λ)

(A5) ei(Cn,sn,x)≜1⌈2nRi⌉⌈2nR˜i⌉∑mi∑m˜iP∥xin(mi,m˜i)+sn+x+NYn−xin(mi′,m˜i′)∥2−−−−−−−−−≤∥sn+x+NYn∥2forsome(mi′,m˜i′)≠(mi,m˜i).

Assume that the receiver has reconstructed ${\left(m_{j},{\tilde{m}}_{j}\right)}_{j\in ⟦1,i⟧}$, for i∈L(Λ). Assume first that $i+1\in I^{c}$. Using minimum distance decoding, on average over the codebooks, we show that the receiver can reconstruct $x_{i+1}^{n}$. We have

$$\begin{array}{cc} \; & E_{C_{n}}\left[e_{i}\left(C_{n},s^{n},\sum_{j=i+1}^{\left|L(\Lambda )\right|}x_{j}^{n}\left(M_{j},{\tilde{M}}_{j}\right)\right)\right] \end{array}$$

(A6a) $$\begin{array}{cc} \; & \leq E_{C_{n}}\left[\left.e_{i}\left(C_{n},s^{n},\sum_{j=i+1}^{\left|L(\Lambda )\right|}x_{j}^{n}\left(M_{j},{\tilde{M}}_{j}\right)\right)\right|C_{n}^{\left(i\right)}\in C_{i}^{*}\right]+P\left[C_{n}^{\left(i\right)}\notin C_{i}^{*}\right] \end{array}$$

(A6b) $$\begin{array}{cc} \; & \overset{n\to \infty }{\to }0, \end{array}$$

where in Equation (A6a) $C_{i}^{*}$ represents all the sets of unit norm vectors scaled by $\sqrt{nP_{i}}$ that satisfy the two conditions of Lemma A1 (in Appendix A), Equation (A6b) holds because $P\left[C_{n}^{\left(i\right)}\in C_{i}^{*}\right]\overset{n\to \infty }{\to }1$ by Lemma A1, and $E_{C_{n}}\left[e_{i}\left(C_{n},s^{n},\sum_{j=i+1}^{\left|L(\Lambda )\right|}x_{j}^{n}\left(M_{j},{\tilde{M}}_{j}\right)\right)|C_{n}^{\left(i\right)}\in C_{i}^{*}\right]\overset{n\to \infty }{\to }0$ by Theorem A1 (in Appendix A) using the definition of $R_{i}+{\tilde{R}}_{i}$ and by interpreting the signal of transmitters in ⟦i+1,|L(Λ)|⟧ as noise.

Similarly, when i+1∈I, using minimum distance decoding, on average over the codebooks, the receiver can reconstruct $x_{i+1}^{n}\left({\overset{˘}{m}}_{i+1},{\overset{˚}{m}}_{i+1}\right)$ with a vanishing average probability of error because ${\overset{˚}{m}}_{i+1}$ is known at the receiver and by definition of ${\overset{˘}{R}}_{i+1}$, hence,

(A7) $$\begin{array}{c} E_{C_{n}}\left[e\left(C_{n},s^{n}\right)\right]\overset{n\to \infty }{\to }0. \end{array}$$

**Equivocation**: We first study the average error probability of decoding $\tilde{m}$ given $\left(z^{n},m\right)$ with the following procedure. From i=|L(Λ)| to i=1, given $\left(z^{n},m\right)$, determine ${\dot{\tilde{m}}}_{i}≜\psi_{i}\left(z^{n},\sum_{j=i+1}^{\left|L(\Lambda )\right|}\sqrt{h_{j}}x_{j}^{n}\left(m_{j},{\dot{\tilde{m}}}_{j}\right)\right)$, where for i∈L(Λ)

(A8) ψi:(zn,x)↦m˜iif∥zn−x−hixin(mi,m˜i)∥2<∥zn−x−hixin(mi,m˜i′)∥2−−−−−−−−−−−−−−−−−−form˜i′≠m˜i0ifnosuchm˜i∈⟦1,2nR˜i⟧exists.

We define $\tilde{e}\left(C_{n}\right)≜P\left[\left.\dot{\tilde{M}}\neq \tilde{M}\right|C_{n}\right]$. We have

(A9) $$\begin{array}{c} \tilde{e}\left(C_{n}\right)=\sum_{i\in L(\Lambda )}{\tilde{e}}_{i}\left(C_{n},\sum_{j=1}^{i-1}\sqrt{h_{j}}x_{j}^{n}\left(M_{j},{\tilde{M}}_{j}\right)\right), \end{array}$$

where for i∈L(Λ)

(A10) e˜i(Cn,x)≜1⌈2nR˜i⌉∑m˜iP∥hixin(mi,m˜i)+x+NZn−hixin(mi,m˜i′)∥2−−−−−−−−−−−−≤∥x+NZn∥2forsomem˜i′≠m˜i.

Similar to the justifications for obtaining Equation (A6b), $E_{C_{n}}\left[{\tilde{e}}_{i}\left(C_{n},\sum_{j=1}^{i-1}\sqrt{h_{j}}x_{j}^{n}\left(M_{j},{\tilde{M}}_{j}\right)\right)\right]$ vanishes to zero as n→∞ by interpreting the signal of transmitters in ⟦1,i−1⟧ as noise and by using the definition of ${\tilde{R}}_{i}$. We thus obtain

(A11) $$\begin{array}{c} E_{C_{n}}\left[\tilde{e}\left(C_{n}\right)\right]\overset{n\to \infty }{\to }0. \end{array}$$

Let the superscript *T* denote the transpose operation and define $X≜{\left[\sqrt{h_{1}}{\left(X_{1}^{n}\right)}^{T}\;\sqrt{h_{2}}{\left(X_{2}^{n}\right)}^{T}\ldots \sqrt{h_{\left|L(\Lambda )\right|}}{\left(X_{\left|L(\Lambda )\right|}^{n}\right)}^{T}\right]}^{T}\in R^{n|L(\Lambda )|\times 1}$, such that

(A12) $$\begin{array}{c} Z^{n}=GX+N_{Z}^{n}, \end{array}$$

with $G≜\left[I_{n},I_{n},\ldots ,I_{n}\right]\in R^{n\times n|L(\Lambda )|}$ and $I_{n}$ the identity matrix with dimension *n*. Let $K_{X}$ denote the covariance matrix of X. Similar to Equation (50), we have

(A13) $$\begin{array}{c} K_{X}=\mathrm{diag}\left(h_{1}P_{1}I_{n},\ldots ,h_{\left|L(\Lambda )\right|}P_{\left|L(\Lambda )\right|}I_{n}\right). \end{array}$$

Then, we have

(A14a) $$\begin{array}{cc} I(M;Z^{n}|C_{n})\; & \leq I\left(X;Z^{n}\right)-H\left(\tilde{M}|C_{n}\right)+H\left(\tilde{M}|Z^{n}MC_{n}\right) \end{array}$$

(A14b) $$\begin{array}{cc} \; & \;\;\;\;\;\;\;\;\;\;\;\;\;\;\;\;\;\;\;\;\;\;\;\;\;\;\;\;\;\;\;\;\;\;\;\;\;\;\;\leq \frac{1}{2}\log\left|GK_{X}G^{T}+I_{n}\right|-H\left(\tilde{M}|C_{n}\right)+H\left(\tilde{M}|Z^{n}MC_{n}\right) \end{array}$$

(A14c) $$\begin{array}{cc} \; & \;\;\;\;\;\;\;\;\;\;\;\;\;\;\;\;\;\;\;\;\;\;\;\;\;\;\;\;\;\;\;\;\;\;\;\;\;\;\;=\frac{n}{2}\log\left(1+\sum_{l\in L(\Lambda )}h_{l}P_{l}\right)-H\left(\tilde{M}|C_{n}\right)+H\left(\tilde{M}|Z^{n}MC_{n}\right) \end{array}$$

(A14d) $$\begin{array}{cc} \; & \;\;\;\;\;\;\;\;\;\;\;\;\;\;\;\;\;\;\;\;\;\;\;\;\;\;\;\;\;\;\;\;\;\;\;\;\;\;\;\;\;\;\;\;\leq nI\left(X_{L(\Lambda )};Z\right)-n(I\left(X_{L(\Lambda )};Z\right)-2|L\left(\Lambda \right)\left|\delta \right)+O\left(nE_{C_{n}}\left[\tilde{e}\left(C_{n}\right)\right]\right) \end{array}$$

(A14e) $$\begin{array}{cc} \; & =2|L(\Lambda )|\delta +o(n), \end{array}$$

where Equation (A14a) holds similar to Equation (51d), Equation (A14b) holds similar to Equation (51f), Equation (A14c) holds by Equation (A13), in Equation (A14d), we used the definition of $\sum_{i\in L(\Lambda )}{\tilde{R}}_{i}$ and the uniformity of $\tilde{M}$ to obtain the second term, and Fano’s inequality to obtain the third term, Equation (A14e) holds by Equation (A11).

The proof of joint secrecy for Step 1 and the repetitions of Step 2 is similar to the proof of Theorem 2.

## Appendix C. Proof of Theorem 5

The proof that Equation (20) is an upper bound on the secrecy sum-rate is similar to the case L=2 in Theorem 3.

Remark that from the statement of Corollary 1, it is unclear whether the sum-rate of Theorem 5 is achievable. However, by inspecting the proof of Theorem 4, observe that we achieve a point in $D_{+}\left(\Lambda \right)≜D\left(\Lambda \right)\cap R_{+}^{\left|L(\Lambda )\right|}$, where D(Λ) is defined in Equation (A2). Hence, the sum-rate of Theorem 5 is indeed achievable.
